# PhoB Activates *Escherichia coli* O157:H7 Virulence Factors in Response to Inorganic Phosphate Limitation

**DOI:** 10.1371/journal.pone.0094285

**Published:** 2014-04-07

**Authors:** Samuel Mohammed Chekabab, Grégory Jubelin, Charles M. Dozois, Josée Harel

**Affiliations:** 1 Research Group on Infectious Diseases of Swine, Montreal University, Faculty of Veterinary Medicine, Saint-Hyacinthe, Québec, Canada; 2 Unité de Microbiologie (UR454) INRA Clermont-Ferrand-Theix, St-Genes-Champanelle, France; 3 INRS-Institut Armand-Frappier, Laval, Québec, Canada; University Medical Center Utrecht, Netherlands

## Abstract

Enterohemorrhagic *Escherichia coli* (EHEC), an emerging food- and water-borne hazard, is highly pathogenic to humans. In the environment, EHEC must survive phosphate (Pi) limitation. The response to such Pi starvation is an induction of the Pho regulon including the Pst system that senses Pi variation. The interplay between the virulence of EHEC, Pho-Pst system and environmental Pi remains unknown. To understand the effects of Pi deprivation on the molecular mechanisms involved in EHEC survival and virulence under Pho regulon control, we undertook transcriptome profiling of the EDL933 wild-type strain grown under high Pi and low Pi conditions and its isogenic Δ*phoB* mutant grown in low Pi conditions. The differentially expressed genes included 1067 Pi-dependent genes and 603 PhoB-dependent genes. Of these 131 genes were both Pi and PhoB-dependent. Differentially expressed genes that were selected included those involved in Pi homeostasis, cellular metabolism, acid stress, oxidative stress and RpoS-dependent stress responses. Differentially expressed virulence systems included the locus of enterocyte effacement (LEE) encoding the type-3 secretion system (T3SS) and its effectors, as well as BP-933W prophage encoded Shiga toxin 2 genes. Moreover, PhoB directly regulated LEE and *stx2* gene expression through binding to specific Pho boxes. However, in Pi-rich medium, constitutive activation of the Pho regulon decreased LEE gene expression and reduced adherence to HeLa cells. Together, these findings reveal that EHEC has evolved a sophisticated response to Pi limitation involving multiple biochemical strategies that contribute to its ability to respond to variations in environmental Pi and to coordinating the virulence response.

## Introduction

Two-component systems (TCSs) are signal transduction pathways commonly used by prokaryotes to sense and adapt to stimuli in the environment; as many as 50 different systems exist in bacteria, and at least 36 known TCSs are used by *E. coli* K-12 [1], [2]. A typical TCS includes a histidine kinase (HK) and a partner response regulator (RR). In response to an input signal, the HK is auto-phosphorylated. Histidine-to-aspartate phosphotransfer to the RR results in transcriptional regulation and a cellular output response.

Inorganic phosphate (Pi) participates in many fundamental cellular processes [3]. In *E. coli*, the Pst system and the PhoB-R TCS coordinately mediate Pi sensing. The Pst system has two related but distinct functions: high-affinity uptake of Pi and the sensing of Pi levels regulating expression of the Pho regulon [4]. The *pst* operon (*pstSCAB–phoU)* encodes a transport complex belonging to the ABC transporter superfamily. The periplasmic protein PstS binds Pi, whereas PstA and PstC form a membrane channel. The ATPase PstB provides the energy for translocation and interacts with PstC [5]. PhoU has no evident role in Pi transport, but is required for control of the Pho regulon. In *E. coli*, there are at least 40 gene members of the Pho regulon that are primarily involved in phosphate assimilation and metabolism [6]–[9]. Transcription of all these genes is regulated in response to extracellular Pi concentrations via the TCS PhoB-R. During Pi limitation, the histidine-kinase PhoR phosphorylates PhoB. This in turn activates Pho regulon expression by binding to specific Pho box sequences located within the promoters of genes belonging to Pho regulon genes [6]. In addition, null mutations in *pst* genes disrupt the regulation of PhoB activation and lead to constitutive expression of the Pho regulon independently of environmental Pi availability [4]. Conversely, when Pi is replete, repression of the Pho regulon is mediated by an interaction between the Pst complex and PhoR, preventing PhoR-mediated phosphorylation of PhoB [10]. In these Pi-rich conditions, expressions of the Pst system becomes repressed and the Pit system induced. Reports indicate that in many bacteria, the Pi-limiting environment or the disruption of the Pst system, induce the Pho regulon and sometimes affect bacterial virulence [11]–[17].

Enterohaemorrhagic *Escherichia coli* (EHEC) serotype O157:H7 is an important pathogen that can cause a variety of clinical symptoms ranging from mild to severe bloody diarrhoea. The main virulence factors of EHEC are Shiga toxins (Stx), responsible for the hemorrhagic syndrome of the infection such as hemolytic uremic syndrome (HUS), and a T3SS through which EHEC translocates effector proteins into host cells, causing intestinal attaching and effacing (A/E) lesions [18], [19]. The genes required for A/E lesions are encoded within a chromosomal pathogenicity island named the locus of enterocyte effacement (LEE) [20]. The LEE is composed of five major operons. The first gene of the LEE1 operon, *ler*, encodes a transcriptional regulator that positively regulates the expression of the LEE2, LEE3, LEE4 and LEE5 operons [21]–[24]. The LEE encodes the T3SS, an adhesin (the intimin Eae) and its receptor (Tir) required for intimate adherence to epithelial cells, and effector proteins translocated through the T3SS into the host cell. The complex regulation of LEE expression involves many positive and negative regulators [25]. Most of these factors influence LEE expression by directly or indirectly controlling *ler* expression; however, a variety of extra-transcriptional mechanisms have also been described that modulate the production of T3SS [26]. In EHEC, the genes encoding Stx1 and Stx2 are located in the genomes of lambdoid bacteriophages that can be induced from lysogenic strains. In the temperate state of the lambda (λ) phage, the CI repressor silences the transcription of most phage genes. The removal of repression that can occur when DNA damage activates the bacterial SOS response, leads to prophage induction [27], [28].

EHEC is widely distributed in domestic ruminants, an important route for transmission to humans [29]. Cattle are the main reservoir for EHEC, especially serotype O157:H7 that is transmitted to humans primarily through consumption of contaminated foods. Faecal contamination of water and other foods may also lead to infection. EHEC can survive and persist in different ecological habitats such as soil, manure and aquatic environment (reviewed in [30]).

We have previously shown that during interaction between EHEC and the predacious protozoa *Acanthamoeba castellanii*, a free-living amoeba mostly found in aquatic environments, the expression of the Pho regulon contributed to adaptation to compete with amoebae in an environment that was limited in nutrients [31]. Indeed in co-culture, the EDL933Δ*pst* strain reduced the growth of *A. castellanii* even more than the wild-type strain. This is also supported by the observation of an increase in expression of Pho genes in strain EDL933 when facing *A. castellanii* [32]. The Pho regulon is a global regulatory circuit involved in bacterial Pi management that has the ability to alter other cellular responses and virulence traits that affect bacterial survival when in association with amoebae in the environment [6], [33]. In *V. cholerae,* the Pho regulon is required for its survival in both fresh water environments and the host small intestine [34].

EHEC, a facultative pathogen, thrives in nutritionally disparate conditions, bovine and human intestines, and in ecological habitats. The essential nutrient Pi is limited in some conditions such as the aquatic ecosystems and terrestrial environments. The concentration of Pi is usually low in natural environments [35], [36]. The availability of Pi in the intestine is variable. It has been estimated from metabolic studies that in healthy adults consuming an average western diet, a Pi concentration of 15 to 30 mM is normally present under homeostatic conditions in the human intestinal lumen [37]–[39]. This level of *in vivo* Pi concentration should repress the Pho regulon. However, high level expression of some Pho regulon members (UgpB, PhoE, PhoA, Pst and PhoU) has been observed in EHEC O157:H7 isolated from piglet gut [40]. This indicates that EHEC cells can encounter Pi starvation signals in open environments as well as *in vivo.* Because we and others have observed that either Pi limitation or inactivation of the Pst system can modulate the virulence of intestinal and extra-intestinal bacterial pathogens [11], [12], [14], [16], [41]–[45], we hypothesized that EHEC virulence factors could be modulated in Pi-limited conditions.

## Materials and Methods

### Bacterial Strains and Culture Media

All strains used in this study are listed in Table 1. We used the EHEC O157:H7 strain EDL933 and its mutants Δ*phoB* and Δ*pstCAB* [31]. To measure the expression level of the *stx2* promoter, a genetic amplifier reporter system was used as described [46]. This amplification system replaced the chromosomal coding sequence of *stx2AB* operon with the coding sequence of the T7 RNA polymerase gene (*T7pol*), resulting in strain EDL933Δ*stx2::T7pol*. This strain was next transformed with the multi-copy reporter plasmid pHL40 [46] that expresses *gfp* under the control of the T7pol-specific promoter. The Gfp background was determined in the EDL933Δ*stx* strain without *T7pol* fusion, also carrying the pHL40 plasmid. The fluorescence emission values (λ 520 nm) were normalized with OD600 nm values. As a positive control for *stx2* transcription, 500 μg/mL of mitomycin C (MitC; the DNA-damaging agent) [47], was added to wild-type strain grown in high Pi during 3 h to induce the BP933W phage [48].

**Table 1:** *E. coli* strains and plasmids used in this study.

Strain/Plasmid	Description and relevant characteristics a	Source reference
EHEC strains		
EDL933WT	*E. coli* O157:H7; wild-type	[80]
EDL933*Δpst*	EDL933; *pstCAB::Km*; Pho-regulon constitutive	[31]
EDL933*ΔphoB*	EDL933; *phoB::Km*; Pho-regulon negative	[31]
EDL933*ΔescN*	EDL933; *escN::Km*; non-functional T3SS mutant	[56]
EDL933Δ*stx*	EDL933; Δ*stx1; stx2::Km*	[55]
EDL933Δ*stx2::T7pol*	EDL933; *stx1::Km*; *stx2::T7pol-Cm^r^*	This study
*E. coli* laboratory strains		
?7213	SM10λpir *ΔasdA4,* Km^r^	[81]
Plasmid		
pAN92	pACYC184::pst operon Cm^r^	[82]
pHL40	Reporter plasmid that expresses *gfp* under the control of a T7 RNA polymerase-specific promoter and Gm^r^	[46]
		
pHSG575	Low-copy-number cloning vector	[83]
pHSG575-*phoBR*	pHSG575, *phoBR* ^+^ Cm^R^ Km^R^	This study

a Km^r^, kanamycin-resistant; Cm^r^, chloramphenicol-resistant; Gm^r^, gentamicin-resistant; Ap^r^; ampicillin-resistant

### Pi Starvation, Culture Experiments and Alkaline Phosphatase Assay

EDL933 strains were grown at 37 °C in 3-(Nmorpholino) propanesulfonic acid (MOPS Teknova) minimal medium [49] supplemented with 0.2% (w/v) glucose, thiamine (0.1 μg/mL), and indicated concentrations of Pi (K_2_HPO_4_). To test bacterial responses to Pi concentrations, cells from overnight LB cultures were inoculated into 25 mL of MOPS medium containing 1.32 mM KH_2_PO_4_ with a starting OD600 nm at 0.15 and grown to an OD600 nm of 0.3. Cells harvested from these fresh cultures were washed once and resuspended in 25 mL MOPS media containing either 1.32 mM (Pi-rich  =  Pi^+^) or 1 μM (Pi-limited  =  Pi^−^) KH_2_PO_4_. Growth was monitored during 16 hours by measuring OD600nm and plating followed by CFU counts. For time-course monitoring of the Pho regulon expression, alkaline phosphatase (AP) activity was measured as described previously [12], [14]. Briefly, aliquots were removed at indicated times, and 4 μg/ml of *p*-nitrophenyl phosphate (Sigma) was added to cells permeabilized by 50 μl of 0.1% SDS and 50 μl of chloroform. Color development was monitored at 420 _nm_ and AP activity was expressed in Miller units (M.U.), and was calculated as follows: 1000 × [OD420 nm - (1.75 × OD550 nm)] / *T* (min) × *V* (ml) × OD600 nm].

In MOPS Pi^−^ medium, the growth curve was similar between the wild-type and Δ*phoB* strains. However, the growth of the wild-type strain was delayed in Pi^−^ compared to in Pi^+^. Therefore, we chose the comparison points based on similar CFU numbers (Figure S1A) and this was monitored by the measurement of PhoA activity (Figure. S1B).

### RNA Extraction, Microarray Experiments and qRT-PCR

The EDL933WT strain and its isogenic Δ*phoB* mutant were grown in Pi^−^ and/or in Pi^+^ conditions until the OD600 nm reached 0.55 to 0.6. Samples equal to 5 × 10^8^ CFU were taken from this mid-log phase and processed for transcriptome analysis. Ten μg of RNAs were extracted and cDNAs were generated using reverse transcriptase. One μg of the fragmented and biotinylated cDNAs was hybridized onto Affymetrix GeneChip *E. coli*. The data were processed using FlexArray software using Robust Multichip Average (RMA) normalization procedure. The levels of transcription obtained from 3 biological replicates of each experimental condition were compared using the EB (Wright & Simon) algorithm. We then conducted comparisons between EDL933 grown in Pi^−^ and in Pi^+^ conditions and between EDL933 and EDL933Δ*phoB* strains both grown in Pi^−^. The differential expression conditions corresponded to a two-fold change (FC) cut-off and adjusted *p-*value < 0.05. Microarray results were validated by triplicate qRT-PCR. Each reaction was normalized to the *tus* housekeeping gene and the variation rate was calculated using the 2^−ΔΔCt^ method. All differential transcripts in both comparisons were functionally classified in COG according to TIGR's *Comprehensive Microbial Resource* [50].

### Production of Constitutively Active PhoB (PhoB^CA^)

The construction of the gene coding for the constitutively active PhoB (PhoB^CA^) protein was based on previous work [51] in which the PhoB^CA^ was obtained by the combination of two substitutions: Asp10Ala and Asp53Glu (DADE). PhoB^CA^ was purified and produced by Genscript as follows. Briefly, the *phoB^CA^* gene was cloned in a modified pGEX vector that contains a thrombin protease recognition site between the Glutathione S-Transferase (GST) tag and a multiple cloning site. Following the elution of PhoB^CA^-GST using Glutathione Sepharose resin, the fused protein was cleaved by thrombin protease to remove the GST affinity Tag. The cleavage reaction was incubated again with Glutathione Sepharose 4B beads and the flow-through containing cleaved PhoB^CA^ was collected. The protein concentration was determined by Bradford protein assay with BSA as a standard, and the purity was about 85% as estimated by densitometric analysis of the Coomassie Blue-stained SDS-PAGE gel.

### Electrophoretic Mobility Shift Assays (EMSA)

The EMSA assay was adapted from previous reports [34], [52]. The EMSA reaction mix consisted of PhoB^CA^ at the desired concentrations (2.5, 5.0, 7.5 or 10.0 μM), 50 nM final probe concentration, 0.1 mg/mL calf thymus DNA and 0.1 mg/mL BSA in EMSA buffer (50 mM NaCl, 20 mM Tris, pH 7.4, 0.02% v/v sodium azide). Reactions were incubated for 30 minutes at 25°C, and then loaded onto an 8% native polyacrylamide gel running at 120 V in 1x TBE buffer. Fluorescent bands were visualized using the Fusion Suite imaging scanner. For each gene tested, the probes were amplified from the EDL933 genome using primers listed in Table S1. The forward primers used to generate all the probes contained 5′ 6-FAM fluorescein tags, to enable gel detection using a chemiluminescence scanner. The probes tested by EMSA assay represent candidate Pho-related virulence gene promoter regions that contain Pho box-sequences identified by *in silico* research of the EDL933 genome. To this end, we created a Pho Box matrix based on 12 Pho box-sequences from the known Pho-dependent genes in EDL933. The matrix frequencies were uploaded into the *Gibbs* algorithm tool at http://ccmbweb.ccv.brown.edu/gibbs/gibbs.html that browses the promoter query (Table S4).

### Polyclonal EspB Antibody Production

We used a polyclonal anti-EspB serum obtained by rabbit immunization performed by Genscript (Piscataway, New Jersey, USA). Briefly, 2 rabbits were injected twice at 14-day intervals with a synthetic EspB antigenic peptide “CRRTQDDITTRLRDI” translated from the EDL933 genome. This peptide sequence was chosen according to its predicted antigenic potency and hydrophilic content, as determined by the Jameson and Wolf algorithm. After the second immunization, the serum titer was 1∶512,000 as tested against the peptide by ELISA assay. The anti-EspB serum was then adsorbed overnight at 4°C, at a 1∶1 volume ratio against HB101 *E.coli* cell extract and incubated at 37°C for 2 hours. Adsorbed material was eliminated by a 45-minute 45,000 rpm ultracentrifugation and 0.05% sodium azide was added to the antibody as a preservative. Unless otherwise indicated, all steps were done at 4 °C to prevent degradation.

### Western-blot Analysis

Western-blot analyses were done as previously described [53], [54]. Twenty ml of filtered culture supernatants that were normalized to CFU and OD_600_ and the total secreted proteins, were precipitated overnight with 10% of trichloroacetic acid (TCA) at 4 °C and then used to measure the secretion of EspB. Protein samples were resuspended in SDS sample buffer, boiled for 5 minutes, run on SDS-PAGE gels and transferred to nitrocellulose membranes. Blots were probed with rabbit polyclonal antiserum against EspB (1∶2000) and then with a goat anti-rabbit HRP-linked secondary antibody (Bio-Rad Laboratories, Hercules, CA). Proteins were detected with ECL-Plus horseradish peroxidase using the Western-blotting detection kit Promega.

### Determination of Stx2 Concentrations by ELISA

The production and release of Stx2B was quantified using the ELISA assay as described elsewhere [55]. The EDL933 strain was grown in MOPS/Pi^+^ or Pi^−^. Its Δ*phoB* derivative mutant and the complemented strain (harboring pHSG575-*phoBR*) were grown in MOPS/Pi^−^. Twenty mL cultures, normalized to OD_600_ 0.6, were harvested and 8 mL of the supernatants were filtered and concentrated twice through a centrifugal filter (3KDa Amicon Ultra-4) for 25 minutes at 3,700 ×*g*. The remaining volumes were designed extra-cellular proteins and used to determine the concentration of secreted Stx2. The bacterial pellets were resuspended in 20 mL PBS, lysed by French press/sonication and then centrifuged for 20 minutes at 12,000 rpm. The lysate supernatants were designated whole cell proteins and used to access the amount of Stx2 production.

### HeLa cell cultures and infections

The HeLa epithelial cell line was maintained in DMEM-HEPES with 10% FBS, 100 U/mL penicillin and 100 μg/mL streptomycin at 37 °C under 5% CO_2_. HeLa cells were seeded into 6-well plates (10^8^ cells/well) and grown for 24 hours. Subsequently cells were washed, and 3 ml of fresh complete medium without antibiotics was added to the wells. Cells were infected with 10^9^ bacteria per well for 5 hours. After 3 washes with PBS, cells were fixed using methanol for 15 minutes and stained with 10% Giemsa solution (Gibco) for 20 minutes. The number of adherent bacteria per 25 cells was determined by direct count under a light microscope (Magnification 640 ×). The TTSS Δ*escN* strain [56] was used as negative control.

### Microarray Accession Numbers

Microarray data have been deposited in the Gene Expression Omnibus database www.ncbi.hlm.nih.gov/projects/geo under accession number GSE50529 [NCBI tracking system #16869052].

## Results

### Effects of Pi Starvation and *phoB* Inactivation on the Transcriptomic Profiles of EDL933

EHEC strain EDL933 was grown in MOPS supplemented with low or high Pi at concentrations, respectively 1.0 μM and 1.32 mM. As shown in Figure S1A, a decrease in growth rate was observed when the Pi concentration was limited to 1 μM. We also investigated the role of PhoB in EDL933 adaptation to Pi limitation using the Δ*phoB* mutant. The growth of the Δ*phoB* strain was similar to that of the wild-type strain in Pi-limited medium (Figure S1A). The alkaline phosphatase activity of PhoA is commonly used to evaluate the activation state of the Pho regulon [16]. As expected, the production level of PhoA in the wild-type strain was negatively correlated with Pi concentration. In addition, PhoA level was decreased over 100-fold in the Δ*phoB* mutant compared with the wild-type strain assayed in Pi-limited medium (Figure S1B). Thus, in the wild-type strain, the Pho regulon is highly activated during growth in Pi-limited medium and this activation is abrogated in the Δ*phoB* mutant.

**Figure 1:**
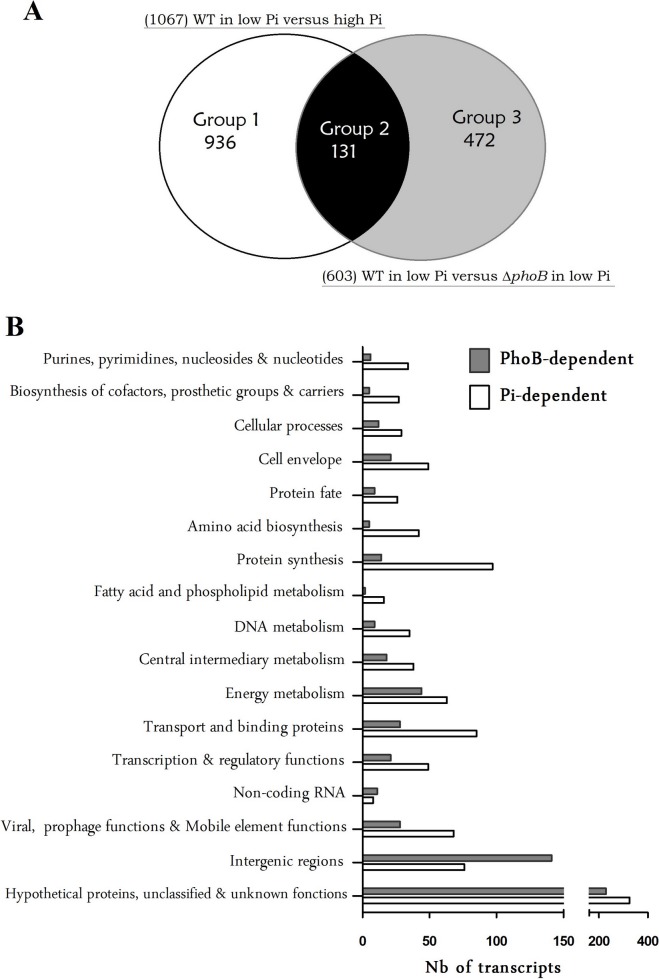
Global analysis of differentially expressed genes in response to Pi starvation or phoB inactivation in EDL933 strain. **A.** Classification of genes whose expression levels were altered in Pi-dependent and in PhoB-dependent manners. Left and right circles indicate the differentially expressed genes of wild-type and Δ*phoB*-mutant strains with expression levels that were altered over 2-fold under Pi limitation. Venn diagram: Group 1 includes 936 PhoB-independent genes that are differentially expressed under Pi limitation in the wild-type strain, but that did not change in the Δ*phoB* mutant. Group 3 includes 472 PhoB-dependent genes differentially expressed in the Δ*phoB* mutant but did not change under Pi-limitation in the wild-type strain. Group 2 included 131 PhoB-dependent Pi response genes that are differentially expressed under Pi limitation in the wild-type strain and between the wild-type and the Δ*phoB* strains. **B.** Functional classification of genes with altered expression in strain EDL933 grown in Pi-limited conditions compared to cells grown in Pi-rich conditions (Pi-dependent (white bars)) and EDL933 incubated in Pi-limited conditions compared to Δ*phoB* mutant cells grown in the Pi-limited conditions (PhoB-dependent (gray bars)).

Transcriptomic analysis was performed to identify the regulatory circuit of PhoB in EDL933. Complementary DNAs obtained from total RNA were hybridized onto Affymetrix GeneChip *E. coli* 2.0 covering the entire EDL933 genome. Compared to the expression profile in high Pi medium, the expression of 1067 genes in the wild-type grown in low Pi medium were changed more than two-fold with an adjusted *P* value of <0.05 (Figure 1A) (Tables S1 and S2). We also compared the transcriptome of WT and Δ*phoB* strains cultivated in low Pi medium and found that 603 genes were differentially expressed.

Combining these two transcriptomic analyses, we classified genes into 3 distinct categories as shown in Figure 1A. Group 1 includes 936 genes with expression levels that changed in the wild-type strain between low and high Pi concentration, but did not change between wild-type and Δ*phoB* strains, therefore corresponding to PhoB-independent Pi response genes. In contrast, Group 3 includes 472 genes with expression levels that changed between wild-type and Δ*phoB* strains, but with no expression change in the wild-type strain grown in low or high Pi media, indicating that expression of these genes is PhoB-dependent, but not modulated by Pi concentration. Lastly, Group 2 includes 131 genes having expression modulated by both Pi concentration and by the PhoB regulatory protein (Table 2). The high proportion of genes that are differentially expressed between the Pi^+^ and Pi^-^ regimes could be slightly affected by the difference in growth rate (Figure S1). To minimize that, equivalent CFU amounts were chosen from Pi^+^ and Pi^−^ conditions that resulted in similar expression patterns of the Pho regulon members presented in Table 2.

**Table 2:** Genes among upregulated and downregulated PhoB-dependent Pi response genes.

Functional class and gene name	Known or predicted function		*WT/P_i_^−^ vs WT/P_i_^+^*		*WT/P_i_^−^ vs Δ*phoB*/P_i_^−^*	
			FC	*P* value	FC	*P* value
DNA metabolism						
*mutY*		Adenine DNA glycosylase	−5.45	2.33E-04	−2.19	1.01E-02
*recG*		ATP-dependent DNA helicase RecG	−3.02	8.04E-03	−3.46	5.12E-03
Purines, pyrimidines, nucleosides. and nucleotides						
*gsk*		Inosine-guanosine kinase	−2.31	4.99E-03	−2.15	9.62E-03
*purT*		P-ribosylglycinamide formyltransferase 2	−3.00	6.16E-03	−2.17	8.97E-03
Energy metabolism						
*ppc*		P-enolpyruvate carboxylase	−3.80	5.54E-04	−2.05	1.02E-02
Central intermediary metabolism						
*phoA*		Alkaline phosphatase (*phoA-psiF*)	131.70	2.48E-07	58.70	3.99E-07
*gadB*		Glutamate decarboxylase (*gadBC*)	10.46	9.58E-05	2.41	1.10E-02
*amn*		AMP nucleosidase	14.54	3.29E-06	5.24	9.48E-04
Cellular processes						
*ahpC*		Alkyl hydroperoxide reductase (*ahpCF*)	6.28	1.00E-03	11.17	1.07E-03
Amino acid biosynthesis						
*trpL*		Trp operon leader peptide	−9.94	1.16E-05	−2.48	4.22E-03
Cell envelope						
*pgaB*		Biofilm adhesin polysaccharide PGA export lipoprotein	6.74	2.47E-04	2.48	3.50E-02
*slp*		OMP induced after carbon starvation	2.85	4.70E-02	2.75	7.20E-03
*fhuA*		Ferrichrome outer membrane transporter	−4.37	6.48E-03	−2.51	1.80E-02
Protein fate						
*hdeA*		Stress response protein acid-resistance protein	4.49	7.13E-03	2.10	4.03E-02
*iraP*		Anti-RssB factor. RpoS stabilzer during Pi starvation; anti-adapter protein	3.96	3.63E-03	2.42	7.74E-03
Protein synthesis						
*yibD*		Predicted glycosyl transferase	80.87	1.56E-06	14.99	5.68E-05
*Z4332*		Cytotoxin Efa-1 involved in posttranscriptional regulation of T3S proteins	3.10	2.31E-02	2.29	9.88E-03
Transcription & regulatory functions						
*phoB*		DNA-binding response regulator (*phoBR*)	4.28	3.25E-03	13.81	4.71E-04
*gadE*		DNA-binding transcriptional activator	7.32	1.72E-03	2.09	4.31E-02
Transport & binding						
*pstS*		High-affinity P-specific transporter (*pstSCAB-phoU*)	47.48	8.46E-06	15.67	5.31E-06
*phnC*		Phosphonate transporter ATP-binding protein (*phnCDEFGHIJKLMNOP*)	81.45	1.45E-06	11.61	7.08E-05
*ugpB*		Glycerol-3-phosphate transporter subunit (*ugpBAECQ*)	7.92	5.46E-04	5.67	1.74E-03
*phoE*		Outer membrane phosphoporin protein E	40.39	1.09E-04	11.72	1.14E-03
*malE*		Maltose transporter periplasmic protein	−2.46	2.31E-02	-2.09	2.20E-02
*fadL*		Long-chain fatty acid outer membrane transporter	−2.66	3.56E-03	-2.38	5.88E-03
Virulence-related determinants						
*espH*		*Z5115* Effector translocated to the host cell membrane by the T3SS. LEE3 operon	2.42	4.89E-03	2.56	9.88E-03
*escD*		LEE-encoded T3SS component	2.17	1.19E-02	2.38	1.43E-02
*espF*		EspF / hypothetical protein. LEE4 operon	2.02	2.79E-02	2.28	3.15E-02
Unknown function. unclassified and hypothetical protein						
*bssR*		Repressor of biofilm formation by indole transport regulation	18.58	1.35E-03	4.86	6.47E-03
*ygaW*		Predicted inner membrane protein. stress-responsive	5.98	2.65E-04	3.43	6.47E-03
*yfiD*		Autonomous glycyl radical cofactor GrcA	5.58	1.29E-02	3.16	1.42E-02
*psiE*		P-starvation-inducible protein PsiE	20.81	1.59E-05	3.10	1.74E-03
*cybC*		Cytochrome b562	3.56	5.29E-04	2.01	1.41E-02
*uspG*		Universal stress protein UP12	6.89	1.81E-04	2.11	1.45E-02
*yebE*		Inner membrane protein. DUF533 family	5.19	5.75E-05	2.34	6.47E-03
*yjeI*		Conserved protein	3.72	3.01E-04	2.65	3.35E-03
*matB*		Cryptic Mat fimbrillin gene	2.60	9.23E-03	2.37	1.23E-02
*ffs*		Unknown non-coding RNA	11.99	4.59E-05	2.19	1.20E-02
*Z2828*		Predicted protein YoaI	5.68	1.61E-03	4.86	1.85E-03
*Z3620*		Hypothetical protein	7.24	4.79E-04	3.80	2.45E-03
*yheT*		Predicted hydrolase	−3.54	2.79E-03	-2.19	1.12E-02
*yijP*		Conserved inner membrane protein	−5.08	1.03E-03	-2.28	4.32E-02
*yejL*		Hypothetical conserved protein. UPF0352 family	−4.00	3.50E-04	-2.44	6.75E-03
*yhcG*		Hypothetical protein	−2.82	4.20E-03	-2.59	6.44E-03
*ybdZ*		Hypothetical protein	−3.74	2.13E-02	-2.76	6.75E-03
*IG*		Intergenic gerion *IG 696357_696735-r*	−2.21	1.61E-02	-2.88	8.87E-03

The Cluster of Orthologous Gene (COG) classification was used to designate genes whose expression was altered during Pi starvation and was under the control PhoB (Figure 1B). Upregulated genes include those encoding proteins of unknown function, transport and binding proteins, energy and central intermediary metabolism, and transcription (Table S2). Genes that were downregulated include the unknown function genes, and those involved with protein fate; protein synthesis; DNA metabolism; purine, pyrimidine, nucleoside and nucleotide pathways; and cell envelope proteins (Table S3). A large number of genes involved in stress responses and hypothetical proteins were also differentially expressed as were some virulence genes. In low Pi, there was a coupling between Pho regulon gene expression and the central pathways of carbon and energy metabolism, many of which are also formal pathways in Pi metabolism.

The expression patterns of the genome-wide transcriptional response that was observed shared similarities with transcriptomic analyses of *E*. *coli* K-12 during Pi starvation, and with the avian pathogen *E.coli* (APEC) and *Citrobacter rodentium* Δ*pst* mutants [7], [11], [12].

The global classification of differentially expressed genes in Pi limitation is shared with the effects of nutrient depletion. Some of these genes coordinate the expression of hundreds of genes across a variety of cellular functions associated with cellular homeostasis and metabolism (Figure 1B). An expression of PhoB regulon genes was effectively induced in the wild-type strain grown in low Pi. In low Pi, the Pho regulon was downregulated in the Δ*phoB* mutant compared to the wild-type strain. Fold change for Pho regulon genes varied from 190.5- to 2.1-fold (Table 2). Besides those, in low Pi the *bssR* gene showed the highest induction (18.6-fold), whereas the greatest repression was observed for the *trpL* gene (9.9-fold) (Tables S2 and S3). Validation of microarray results was achieved using qRT-PCR. Comparison of gene expression by microarray hybridizations and qRT-PCR demonstrated a high level of concordance between the datasets, which is represented by a correlation coefficient of 0.87 in response to low Pi and of 0.82 in response to Δ*phoB* mutation (Figure S2).

Globally, the transcriptional profile of EDL933 grown in Pi-limited medium indicates that, in addition to up-regulating genes associated with scavenging pathways for Pi acquisition and conservation, many genes dealing directly with global stress were differentially expressed. Many lines of evidence suggest that the Pho regulon and the stress response are interrelated [33]. The Pho regulon and the RpoS regulon are interrelated regulatory networks of the bacterial adaptive response [33]. RpoS is a sigma factor implicated in the cellular response to many stresses. It is also implicated in the stationary phase and the induction of genes in nutrient-limiting environments [57]. The *rpoS* gene is expressed under a variety of growth conditions, but regulation and RpoS production is largely dependent on post-transcriptional stability [57], [58]. In our study, the *rpoS* gene was not differentially expressed in strains grown in Pi-limited medium. However, in this condition the RpoS-regulatory gene *iraP* was induced (4-fold). IraP encodes an anti-adaptor protein that enhances RpoS stability and accumulation by inhibiting its targeting to the ClpXP degradosome [59], [60]. Moreover, Mandel et *al.,* has shown that RpoS translation and stability were significantly increased during Pi starvation [61]. This suggests that in our results the RpoS stability was probably increased in EDL933 grown in Pi-limited medium that could lead to regulatory expression of numerous RpoS-dependent genes. In addition, some genes involved in the oxidative stress and acid stress responses were found to be differentially expressed. Of these, some were modulated in the Δ*phoB* mutant such as *iraP, katE, ahpC, gadE* and *gadBC.* These stress responses were also observed in the Δ*pst* mutant of APEC and *C. rodentium* strains [11], [12].

### Pi-limited Conditions Increased LEE Gene Expression in PhoB-dependent and Independent Manners

**Figure 2:**
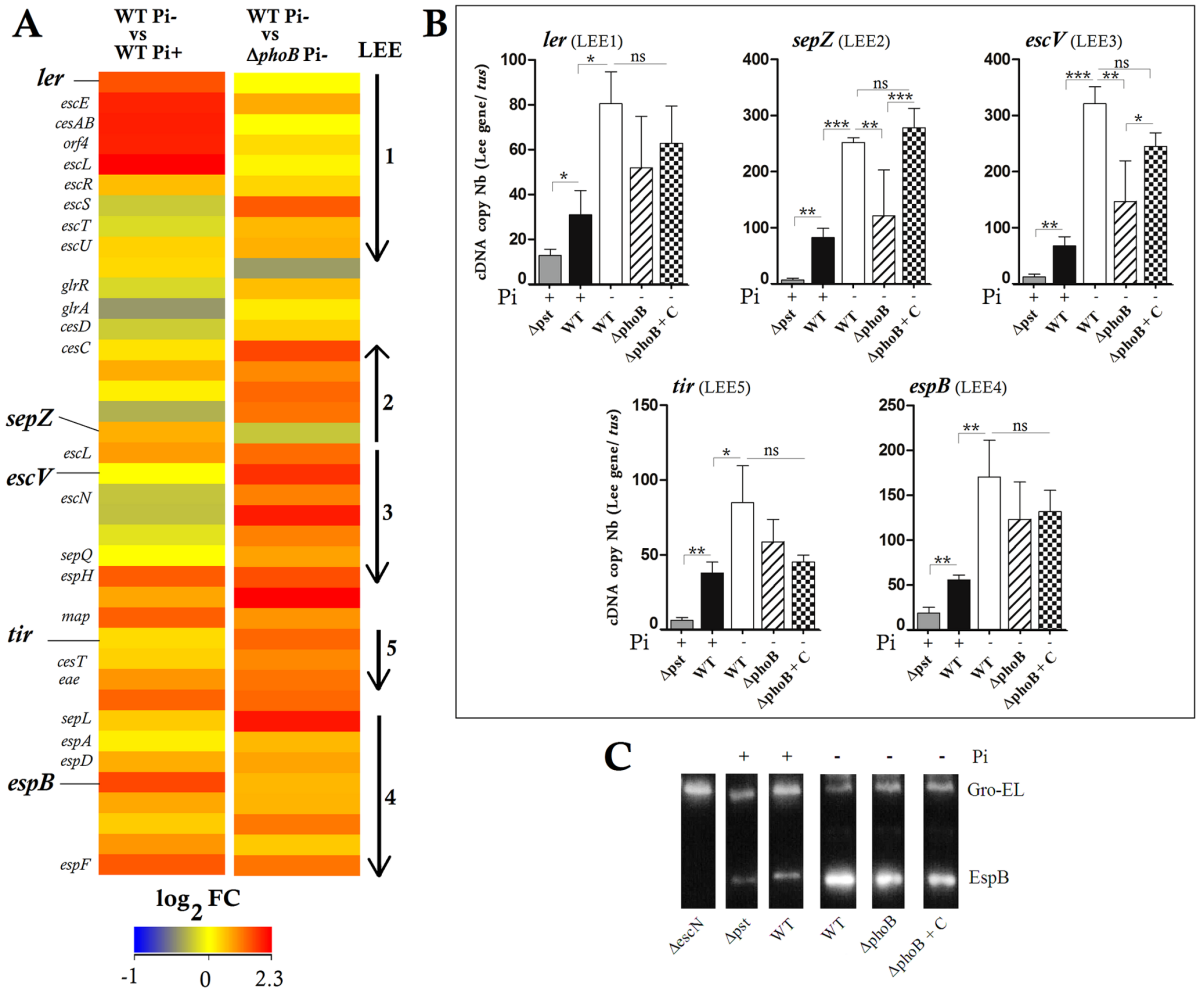
Low Pi conditions and deletion of *phoB* gene increased LEE gene expression and T3SS secretion of EspB in EDL933 strain. A. A heat map of the expression levels of LEE genes between wild-type strain grown in low or high Pi concentrations, and between wild-type and Δ*phoB* strain grown in low Pi condition. Expression values were determined from the variance analysis by the EB (Wright & Simon) algorithm and are represented colorimetrically, with red representing up regulation (ratio of +2.3) and blue representing downregulation (ratio of -1) on a log_2_ scale. The data are represented as the means from three biological replicates. B. Expression level of LEE genes *ler, sepZ, escV, tir* and *espB* was analyzed by RT-qPCR in the wild-type, the Δ*phoB* mutant and its complement (Δ*phoB*+C) and the Δ*pst* mutant grown in low or high Pi media as indicated. C. Western-blot using anti-EspB antibody against the supernatant of the wild-type, the Δ*phoB* mutant and its complement and the Δ*pst* mutant grown in low or high Pi media as indicated. The Δ*escN* mutant grown in DMEM was used as a negative control. One μg/mL of Gro-El was added as a protein precipitation control. Asterisks represent the significant ANOVA *p* value (*<0.05, **<0.01, ***<0.001). ns: Not significant.

EHEC-specific virulence-associated genes were modulated in Pi-limited medium and in the Δ*phoB* mutant. Genes from LEE operons were 2.0- to 4.6-fold upregulated in the wild-type strain grown in Pi-limited medium compared to the wild-type strain grown in Pi-rich medium, and compared to the Δ*phoB* mutant (Fig. 2A). Moreover, several genes encoding LEE regulators such as *gadE* (+7.3), *pchA* (+7.6) and *ihf* (+2.6) were also upregulated in the wild-type strain in response to Pi starvation. However, in the wild-type strain compared to the Δ*phoB* mutant, genes encoding the non-LEE effectors NleB, NleE, NleH and NleG-1 and the LEE-negative regulator HN-S were upregulated (Tables S2).

We quantified the transcripts of *ler, sepZ, escV, espB* and *tir* encoded by LEE1, 2, 3, 4 and 5 respectively in the wild-type strain grown in Pi-limited and Pi-rich media and in Δ*phoB* strain grown in Pi-limited conditions. LEE-gene expression in the wild-type strain was 2.0-fold to 4.6-fold higher in response to Pi limitation (Fig. 2B). The tested genes that were downregulated in the absence of PhoB, *sepZ* and *escV* were the most significantly affected while the complementation of the Δ*phoB* mutant restored the *sepZ* and *escV* transcripts to wild-type levels (Fig. 2B). This suggests that PhoB is at least partially required for LEE activation in low Pi.

The EDL933 strain cultivated in Pi-limited medium delivers higher level of extracellular proteins compared to when grown in Pi-rich medium (data not shown). Similarly, secretion of the effector protein EspB, encoded by LEE4, was more abundant in the wild-type strain grown in low Pi medium (EspB/GroEL ratio  =  3.6 ±0.3) than in high Pi medium (1.2 ±0.3) and to a lesser extent in the Δ*phoB* mutant in Pi-limited medium (2.6 ±0.2) (Figure 2C). The Δ*phoB*-complemented strain showed a slight increase of EspB (2.8±0.4) compared to the mutant. These results showed that higher level of the EspB effector as detected in Pi-limited conditions, confirming that Pi limitation can increase LEE gene expression. Moreover Pi-dependent activation was partially PhoB-dependent.

### In Pi-limited medium, PhoB Activated the Transcription, Production and Release of Stx2 and Controlled Gene Transcription of BP933W Regulators

**Figure 3:**
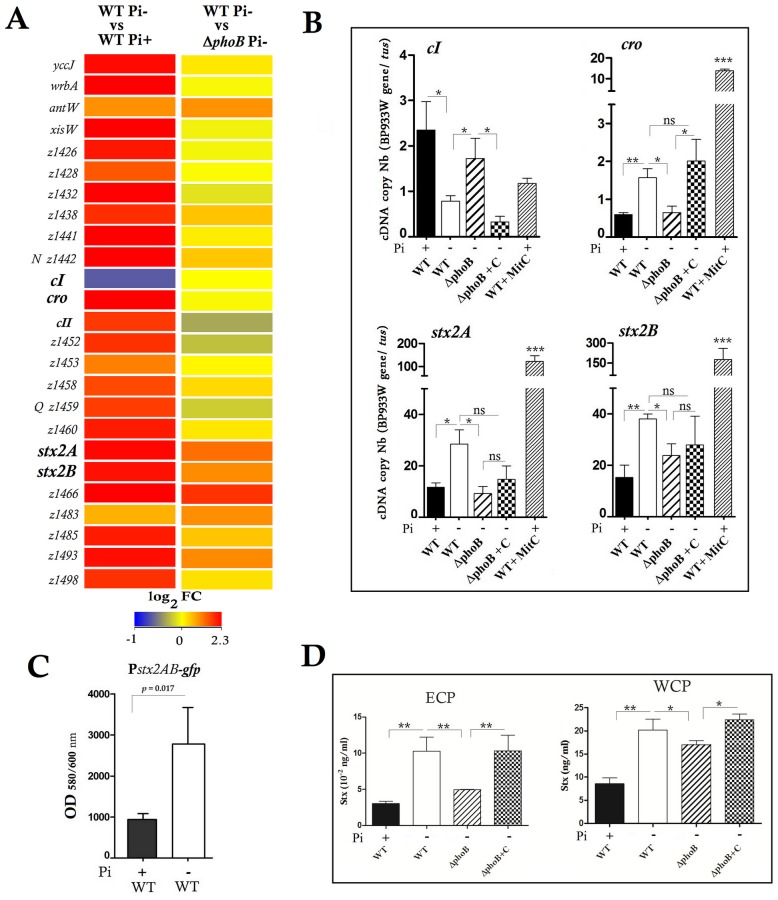
Effect of Pi and PhoB on *stx2* gene expression and toxin production. Low Pi and PhoB increased transcription of *stx2* and its toxin production and of BP933W genes except for repressor gene *cI* that was repressed. **A**. Heat map of the expression levels of BP933W genes between wild-type EDL933 strain grown in low or high Pi conditions and between wild-type and Δ*phoB* strain grown in low Pi conditions. **B.** The expression levels of the BP933W genes *cI, cro* and *stx2AB* were analyzed by RT-qPCR in the wild-type strain and the Δ*phoB* mutant grown in low or high Pi media as indicated. The wild-type strain induced by mitomycin C (WT+MitC) was used as a positive control **C.** Fluorescence of the wild-type EDL933 strain carrying a chromosomal fusion reporting *stx2* transcription level grown in Pi+ or Pi- conditions. **D.** The production of Stx2 measured by ELISA in extra-cellular protein (ECP) and whole cell protein (WCP) fractions of the EDL933 wild-type strain grown in Pi+ or Pi- conditions and in the Δ*phoB* mutant and its complemented derivative. Asterisks represent the significant ANOVA *P value* (*<0.05, **<0.01, ***<0.001).

Many BP-933W phage genes were upregulated in response to Pi starvation, including the anti-terminators N and Q, and the *stx2* and phage-late genes while expression of the CI repressor gene was downregulated (Figure 3A, Tables S2 and S3). In these Pi-limited conditions, genes encoding RecA and LexA, the SOS response marker and regulators, were not differentially expressed, while a gene encoding an indirect λ-phage regulator PcnB was repressed (Table S3).

In EDL933, Stx2 is encoded by *stx2AB* genes in the λ-like lysogenic phage BP933W. The *stx2AB* operon is located downstream of the late promoter region and upstream of the phage lysis cassette [62]–[64].

Measurement by qRT-PCR confirmed the significant decrease of *cI* expression and increase of *cro* and *stx2AB* genes in Pi-limited medium and in a PhoB-dependent manner (Figure 3B). As a positive control, MitC-induction of the BP933W phage resulted in high expression of *cro* and *stx2* genes while *cI* repressor gene expression remained at a basal transcription level. The Δ*phoB*-complemented strain exhibited similar levels of *cI* and *cro* transcription as the wild-type, although expression of the *stx2* genes was only partially restored. The transcriptional activation of *stx2* expression in Pi-limited medium was also observed using a GFP reporter system (Figure 3C).

To evaluate Stx2 synthesis, amounts of Stx2 were quantified in cells and supernatants using an ELISA assay. In response to Pi-limited conditions, the production and release of Stx2B were increased by 2.9-fold (Figure 3D). By contrast, Stx levels were decreased 2-fold in the Δ*phoB* mutant, whereas complementation of the Δ*phoB* mutant restored Stx2 level to that of the wild-type parent strain. These results demonstrate that the production of Stx2 toxin by the EDL933 strain is modulated by Pi levels and the PhoB regulatory protein.

### PhoB Binds to LEE1, LEE2 and *stx2* Promoter Regions *in vitro*

**Figure 4:**
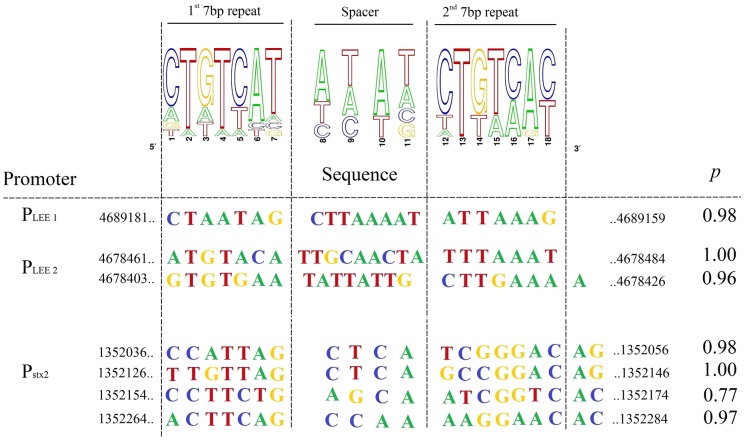
Bio-informatic analyses of PhoB-binding sites. Sequence logos determined from 12 putative PhoB-binding sites in EDL933 are indicated (upper panel). Potential consensus sequences identified in the promoter regions of LEE 1, LEE 2 operons and upstream *stx2AB* genes are shown with their statistical scores and genomic positions (lower panel). Pho box prediction probabilities were determined using the matrix frequencies of Table S4 that were uploaded into the Gibbs software algorithm. http://ccmbweb.ccv.brown.edu/gibbs/gibbs.html.

The PhoB binding consensus sequence is defined as a 22-bp region of double-stranded DNA that consists of two direct repeats of 11 bp [65]. We performed *in silico* analysis to identify putative PhoB-binding sites associated with the LEE promoters (Figure 4). Pho box consensus sequences with high probability were identified in the LEE1 and LEE2 promoter regions. In LEE1, a Pho box was located between the *ler* distal P1 and proximal P2 promoters [66] and two Pho boxes were identified in the LEE2 promoter region [67].

**Figure 5:**
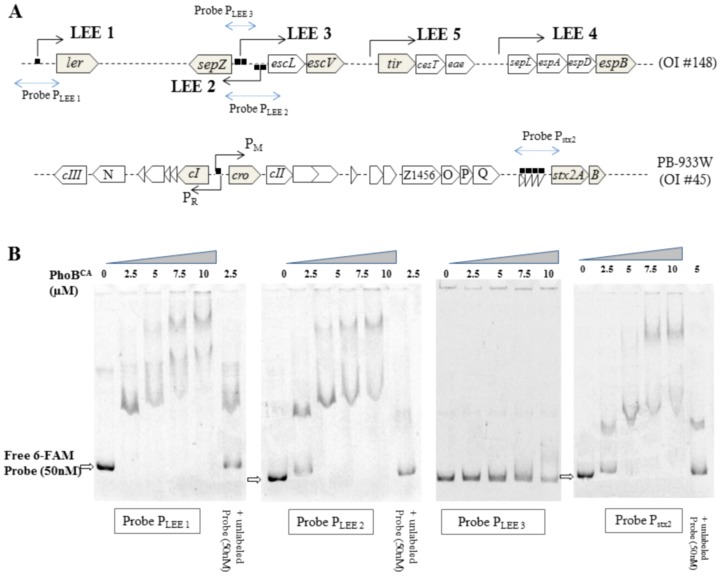
PhoB binds in vitro to LEE1, LEE2 and *stx2* promoter regions. A. Schematic representation of LEE and lambdoid prophage BP933W DNA regions. Arrows indicate the orientation of transcription. The blue lines/arrows indicate the probes used for EMSA assays. The square symbol indicates the predicted Pho box. B. Increasing amounts of GST-purified recombinant PhoB^CA^ were used in the EMSA assay to shift the 6-FAM labeled DNA probes amplified from LEE1 (−253 to +64 bp), LEE2 (−228 to +81 bp), LEE3 (−109 to −259 bp) and *stx2AB* (−402 to −3 bp) promoter regions.

To investigate whether PhoB directly binds to the LEE promoters containing putative PhoB boxes, EMSA experiments were performed using PhoB^CA^, a constitutively active PhoB mutant that activates Pho regulon promoters in the absence of phosphorylation. EMSA results showed PhoB dose-dependent shifts of the probes designed from the LEE1 and LEE2 promoter regions but not the LEE3 promoter region (Figure 5). As expected, the addition of unlabelled probes (1∶1) reduced the amount of shifted 6-FAM labelled probes. This data indicates that PhoB^CA^ binds specifically to the LEE1 and LEE2 promoter regions *in vitro*.

Four Pho box consensus sequences were found in the promoter of the *stx2AB* operon (Figures 4 and 5A). Using PhoB^CA^, EMSA experiments demonstrated that PhoB was able to bind the *stx2AB* promoter region (Figure 5B). Indeed, the probe designed from the *stx2* promoter region shifted with a dose-dependent addition of PhoB^CA^ and the addition of unlabelled control probe competed well with the 6-FAM-labelled probe. Although a potential Pho box was identified in the intergenic region between *cI* and *cro* that overlaps the P_R_ promoter, no shift was observed by EMSA (data not shown). Thus, the increased expression of *stx2* could be a direct effect of PhoB in Pi-limited medium. This would suggest that increased transcription of BP933W genes could result from direct and indirect effects of PhoB, and could also be due to the PhoB-independent effect of Pi-limited conditions.

### Constitutive Activation of the Pho Regulon Decreased LEE Gene Expression and Reduced Adherence of strain EDL933 to HeLa Cells

To investigate the influence of the Pho regulon and PhoB on adherence, we evaluated the attachment of Δ*pst* and Δ*phoB* mutants to HeLa cells in DMEM cell culture medium, which is a Pi-rich medium. The Δ*pst* mutant was used because the inactivation of the Pst system results in constitutive expression of the Pho regulon. Thereby, the Δ*pst* mutant should mimic the behavior of the wild-type strain grown in Pi-limited conditions. Accordingly, the EDL933 Δ*pst* mutant grown in Pi-rich medium demonstrated strong Pho regulon activation as indicated by PhoA activity (350 M.U.) (data not shown). However, expression of LEE genes in the Δ*pst* mutant was decreased, which was in contrast to the wild-type strain in response to Pi starvation, a condition that activates the Pho regulon (Figure 2B). Furthermore, secretion of the EspB effector protein was also reduced in the Δ*pst* mutant confirming the repression of LEE genes in this mutant (Figures 2B and 2C).

**Figure 6:**
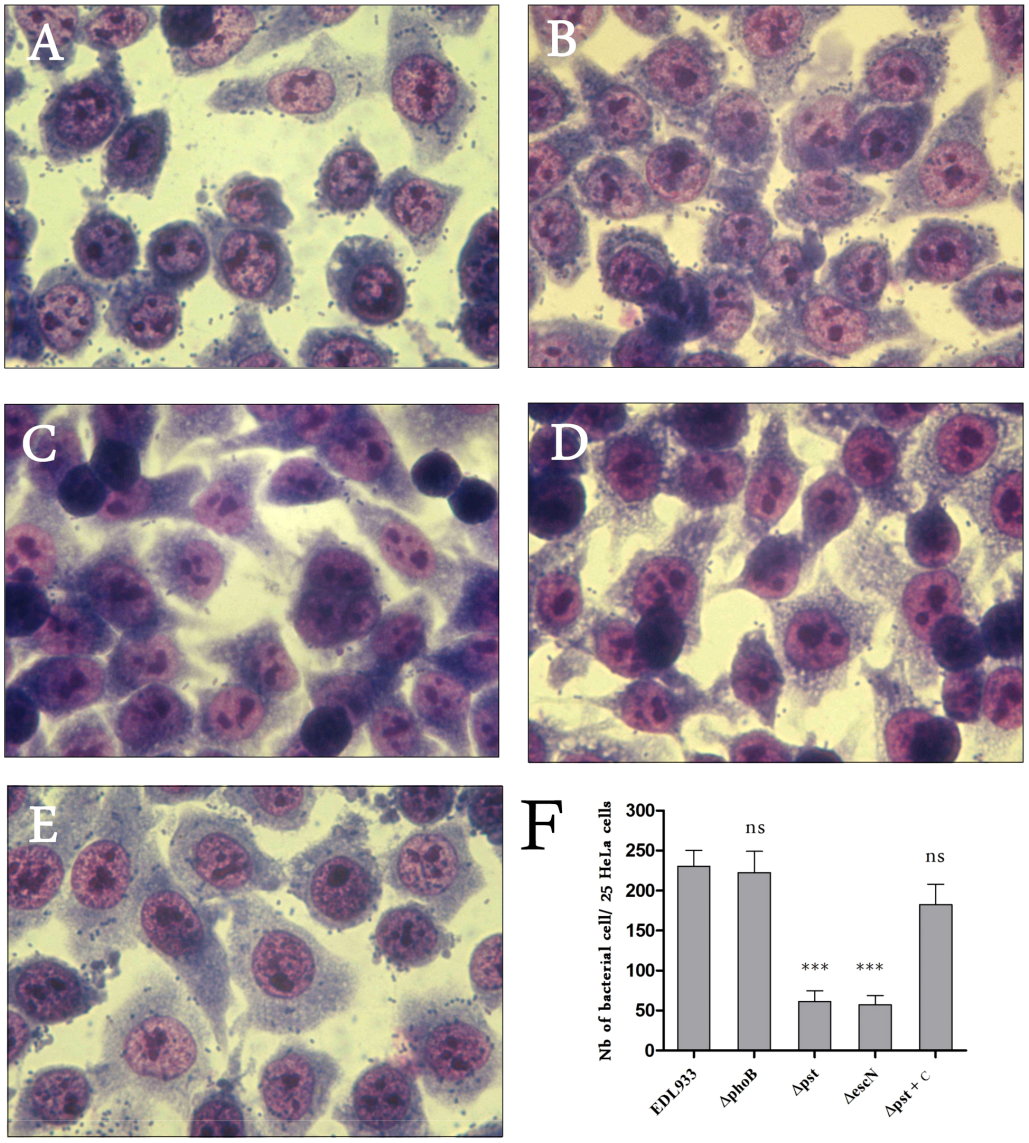
Regulation of adhesion of EDL933 to human epithelial cells by the Pho regulon. HeLa cells were infected with EDL933 and indicated mutants. After 6-type and Δ*phoB* strains (**A** and **B**). The adhesion decreased in the Δ*pst* mutant (*P* <0.001) to levels similar to that of the Δ*escN* mutant which lacks functional TTSS (**C** and **D**). The attachment phenotype was restored in the *trans* complemented Δ*pst* mutant (**E**). The number of adherent bacteria per HeLa cell was determined from 25 cells (**F**). Magnification 64 ×.

While the adhesion phenotype of the Δ*phoB* mutant to HeLa cells was similar to that of the wild-type strain (≈ 230 bacteria/25 HeLa cells) (Figure 6), the adhesion of the Δ*pst* mutant was reduced to a level similar to a Δ*escN* mutant, which lacks a functional T3SS (≈60 bacteria/25 HeLa cells) (Figure 6). Complementation of the Δ*pst* mutant restored the adhesion phenotype of the wild-type parent strain. With regards to LEE gene expression, our results indicate that a Δ*pst* mutant, in which the Pho regulon was highly and unduly activated (*phoB*/*tus* transcript ratio ≈ 127), did not reflect the behaviour of the wild-type strain responding to low Pi in which the Pho regulon was moderately activated (*phoB*/*tus* transcript ratio ≈ 10.5). Nonetheless, LEE gene expression and EHEC adherence properties are importantly affected in a Δ*pst* mutant, indicating that alteration of the Pho regulon modulates the transcription levels of genes belonging to this pathogenicity island.

## Discussion

In response to Pi limitation, EHEC uses the Pho regulon, a specific regulatory network for phosphate acquisition. The two-component signal transduction system of PhoB-R plays a crucial role in inducing Pho regulon genes to adapt to Pi starvation. Herein, we show that during its adaptation to Pi limiting-conditions, EHEC EDL933 modulates gene expression associated with the acquisition and metabolism of Pi and with mechanisms of general stress responses. In addition, concomitant changes in expression of virulence genes occur. This is also observed in other pathogenic *E. coli* including *C. rodentium* that causes EHEC-like intestinal A/E lesions [11]. Changes in global gene expression underlying the overall physiological state of the cells in Pi limitation are also shared with other conditions of nutrient depletion. Some gene subsets responded particularly to Pi limitation stress. The Pho regulon has been shown to contribute to the virulence of atypical enteropathogenic *E. coli* (EPEC) and *C. rodentium* through the response regulator PhoB [11], [44].

During growth under Pi limitation, the absence of PhoB affected the expression of genes involved in Pi metabolism and genes involved in stress response and virulence. This indicates that PhoB plays an important role in the survival of EHEC under stressful Pi limiting conditions. Additionally, we show that PhoB is involved in EHEC virulence by regulating the expression of important virulence genes of the LEE and Stx2. Thus, we identified a novel role for PhoB as a transcriptional regulator of importance for both, adaptation to Pi availability and regulation of key virulence systems of EHEC.

Using transcriptional analysis and ELISA assays we showed that Pi-limiting conditions activate the expression and production of virulence genes encoding the LEE T3SS and some effectors and the Stx2 Shiga toxin. By regulating LEE and Stx2 expression, EHEC is able to control expression of major virulence genes under Pi limiting-conditions. The virulence modulation is partly due to PhoB, or PhoB-regulated gene(s), because a Δ*phoB* mutation affected the expression of some virulence genes in Pi-limiting conditions.

Microarray and qRT-PCR analysis demonstrated for the first time that low Pi-induced signals activated LEE gene expression. Both, low Pi and PhoB increased protein secretion by T3SS. PhoB was required to significantly increase the transcription of LEE2 and LEE3 while the transcription of LEE1 required a low Pi signal rather than PhoB. However, PhoB interacts directly with the promoters of LEE1 and LEE2 operons containing Pho boxes.

The regulation of LEE expression is complex and involves many positive and negative regulators [25]. Most of these factors influence LEE expression through direct or indirect control of *ler* expression. However, a variety of extra-transcriptional mechanisms have also been described that regulate LEE expression [26]. Our results suggest that PhoB could act on LEE gene expression through its binding to the *ler*/LEE1 promoter, which in return would activate the whole LEE. In addition, PhoB is involved in expression of LEE2 by direct interaction with its promoter region. However, it also possible that LEE regulators other than PhoB may also be involved in the fine tuning of expression of LEE genes. Indeed, some positive regulators of LEE such as PchA, IHF and the LEE-negative regulator GadE were found to be upregulated in Pi-limited conditions. In addition, the silencer HN-S was repressed in the Δ*phoB* mutant.

In contrast, the EHEC Δ*pst* mutant showed a reduced expression of LEE and reduced adherence. This is also observed in *C. rodentium* that also harbors the LEE locus, where the expression of some T3SS genes and the ability to adhere and colonize were reduced in the Δ*pst* mutant [44]. In the Δ*pst* mutant, the Pho regulon is constitutively and more strongly activated than in Pi-limiting condition. Indeed, strong activation of the *phoB* gene was found in the Δ*pst* background compared to in the wild-type strain in low Pi. This may reflect the super-shift in EMSA LEE1 and such high levels of phospho-PhoB may actually repress the expression of LEE genes. We have previously shown that the level of activation of the Pho regulon influenced the degree of attenuation of APEC [16]. Moreover, it has been shown recently that different Pi conditions have conflicting requirements of PhoB expression levels for optimal cell fitness [68], [69]. Thus, our results suggest that the level of Pho regulon activation influences expression of LEE. In low Pi conditions, LEE is activated, while in the Δ*pst* mutant, LEE is repressed.

In the Sakai strain, Yoshida *et al.* [70], have demonstrated that the gene cluster esc0540–0544 is positively regulated by PhoB. This gene cluster is homologous to the *siiCA-DA* operon of *Escherichia fergusonii*, which encodes a putative RTX toxin and its cognate type I secretion system (T1SS) [70]. These findings taken together with our results suggest that PhoB acts as a virulence gene regulator in EHEC. Moreover, maintenance of proper regulation is critical, since both underexpression and overexpression can affect its virulence functions.

Signals from the host may arise within the intestine, such as changes in metabolite concentration, which allow bacteria to monitor infection and alter their behavior. Pieper *et al.* [40], have recently shown the activation of the Pho regulon of EHEC in the intestinal environment of gnotobiotic piglets. The signal that leads to induction of the Pho regulon is likely to be Pi limitation. However, we cannot be certain as PhoB can be regulated by a number of signals in addition to Pi concentration [3], [71]–[73].

Many genes of the lysogenic λ phage BP933W were upregulated in response to Pi-limiting conditions including *stx2 w*hile the CI repressor gene was inhibited. The repressor system of BP933W maintains the prophage in a quiescent state, [74]. Phage induction is a complex process dependent on bacterial and phage factors. Prophage induction, which leads to lytic growth, results from removal of repression. Ultimately the repressor controls Stx production and/or release. Stx2 production was increased in Pi-limited medium. Factors that regulate the switch between lysogeny and lytic growth, e.g., repressor, operator sites, and associated phage promoters, play important roles in regulating the production and/or release of Stx [64]. In Pi-limited conditions, PhoB indirectly inhibits the transcription of the repressor gene *cI*, which in return releases the constitutive transcription of *cro* that should induce a lytic phage cycle, thereby increasing *stx2* transcription. Prophage induction ultimately results in Q-modified transcription initiating at P_R′_ that transcends the t_R′_ terminator, leading to expression of downstream genes that include *stx2* and those encoding lysis functions. Another scenario could be direct PhoB binding onto sequences upstream of the *Stx2* promoter that activates *stx2* transcription.

Induction of the SOS response that commonly occurs through damage to the bacterial cell's DNA can lead to significantly higher levels of prophage induction. The SOS response refers to the production of a large number of enzymes following damage to DNA [75]. These enzymes include the activated form of RecA protein that facilitates auto-cleavage of many phage repressors and the bacterial LexA protein, which itself controls expression of the SOS response. In Pi-limited conditions, it appears that phage induction is RecA- and LexA-independent. RecA-independent induction also occurs in phages infecting *E. coli* [76]–[78]. In phage λ, induction does not always imply repressor cleavage by RecA. In some cases, the mechanism involves RcsA, a regulator of colanic acid synthesis, and DsrA [76], [78], a small regulatory RNA that, among other functions, prevents the degradation of RcsA. Imamovic and Muniesa [79] showed that the Mg^++^ chelator EDTA affects the membrane structure of bacteria and triggers λ phage by RecA-independent mechanisms.

All together, these findings reveal that EHEC has evolved a sophisticated response to Pi availability involving multiple biochemical strategies that are likely critical to its ability to respond to changes in environmental Pi levels and also coordination of its virulence gene response by inducing LEE and *stx2* genes and controlling BP933W expression.

## Supporting Information

Figure S1 **A.** Growth (CFU/mL) of EDL933 the WT strain and its isogenic mutants Δ*phoB* in MOPS medium supplemented with 1.32 mM (Pi+) or 1.0 μM (Pi-) KH_2_PO_4_ for 16 h at 37°C. **B.** PhoA activity monitoring during 6 h cultivation of EDL933 in Pi+ or Δ*phoB* in Pi- leads to abolition of the Pho regulon while it is activated in WT grown in Pi-. Arrows indicate the 2 comparisons points chosen for the rest of the experiments in this study.
(DOCX)

Figure S2 **Microarray results were validated by triplicate qRT-PCR on 13 representative genes.** Each reaction was normalized to *tus* gene and the variation rate was calculated using 2^-ΔΔCt^ method.
(DOCX)

Table S1 **Primers used in this study.**
(DOCX)

Table S2 **Functional classification of upregulated genes of microarray data comparing the wild-type strain grown low Pi to in high Pi (Pi-dependent) and comparing the wild-type strain to Δ** ***phoB*** **mutant both grown low Pi (PhoB-dependent).**
(DOCX)

Table S3 **Functional classification of downregulated genes of microarray data comparing the wild-type strain grown low Pi to in high Pi (Pi-dependent) and comparing the wild-type strain to Δ** ***phoB*** **mutant both grown low Pi (PhoB-dependent).**
(DOCX)

Table S4 **Pho Box matrix based on 12 Pho-Boxes sequences from the known Pho genes in EDL933 identified by Yuan** ***et al*** **. 2006.**
(DOCX)
