# Supplementary material for: PhoB Activates Escherichia coli O157:H7 Virulence Factors in Response to Inorganic Phosphate Limitation
Source: PLoS One. 2014 Apr 7;9(4):e94285. doi: 10.1371/journal.pone.0094285 (PMC3978041; doi:10.1371/journal.pone.0094285)
Supplement: Figure S1 — A. Growth (CFU/mL) of EDL933 the WT strain and its isogenic mutants ΔphoB in MOPS medium supplemented with 1.32 mM (Pi+) or 1.0 μM (Pi-) KH2PO4 for 16 h at 37°C. B. PhoA activity monitoring during 6 h cultivation of EDL933 in Pi+ or ΔphoB in Pi- leads to abolition of the Pho regulon while it is activated in WT grown in Pi-. Arrows indicate the 2 comparisons points chosen for the rest of the experiments in this study. (DOCX) [file pone.0094285.s001.docx]

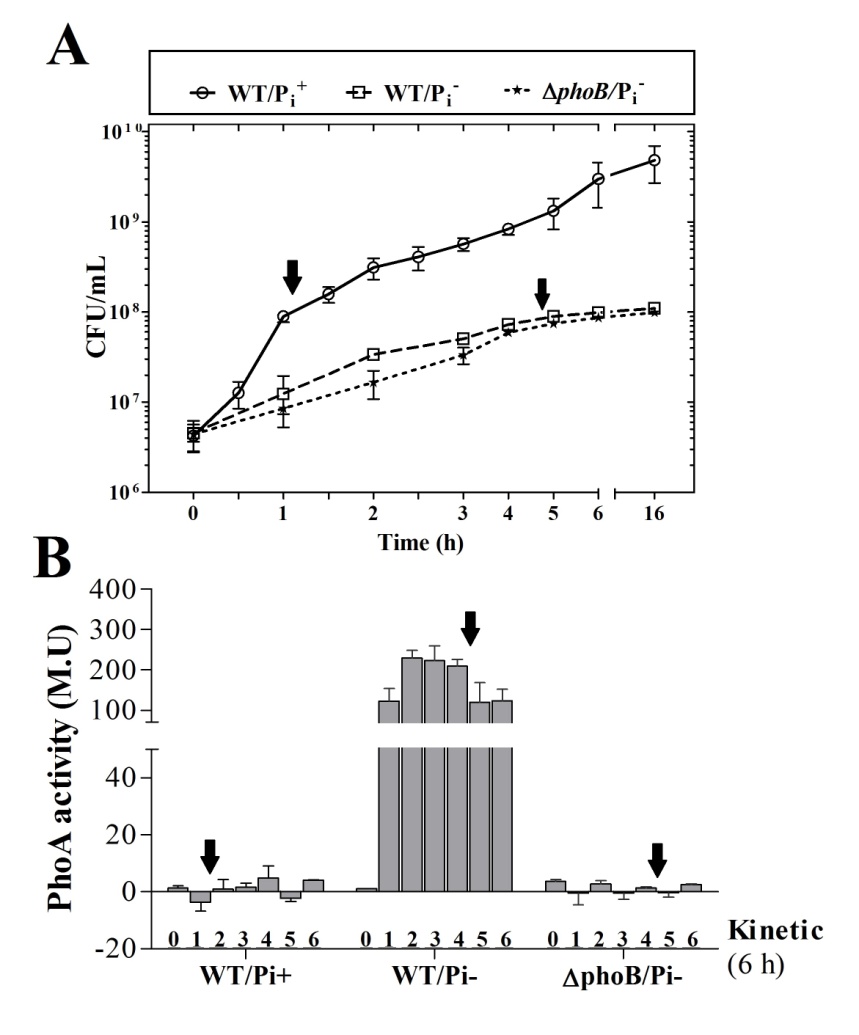


**Figure S1.** **A.** Growth (CFU/mL) of EDL933 the WT strain and its isogenic mutants Δ*phoB* in MOPS medium supplemented with 1.32 mM (Pi+) or 1.0 μM (Pi-) KH_2_PO_4_ for 16 h at 37°C. **B.** PhoA activity monitoring during 6 h cultivation of EDL933 in Pi+ or Δ*phoB* in Pi- leads to abolition of the Pho regulon while it is activated in WT grown in Pi-*.* Arrows indicate the 2 comparisons points chosen for the rest of the experiments in this study.
