# Supplementary material for: PhoB Activates Escherichia coli O157:H7 Virulence Factors in Response to Inorganic Phosphate Limitation
Source: PLoS One. 2014 Apr 7;9(4):e94285. doi: 10.1371/journal.pone.0094285 (PMC3978041; doi:10.1371/journal.pone.0094285)
Supplement: Figure S2 — Microarray results were validated by triplicate qRT-PCR on 13 representative genes. Each reaction was normalized to tus gene and the variation rate was calculated using 2-ΔΔCt method. (DOCX) [file pone.0094285.s002.docx]

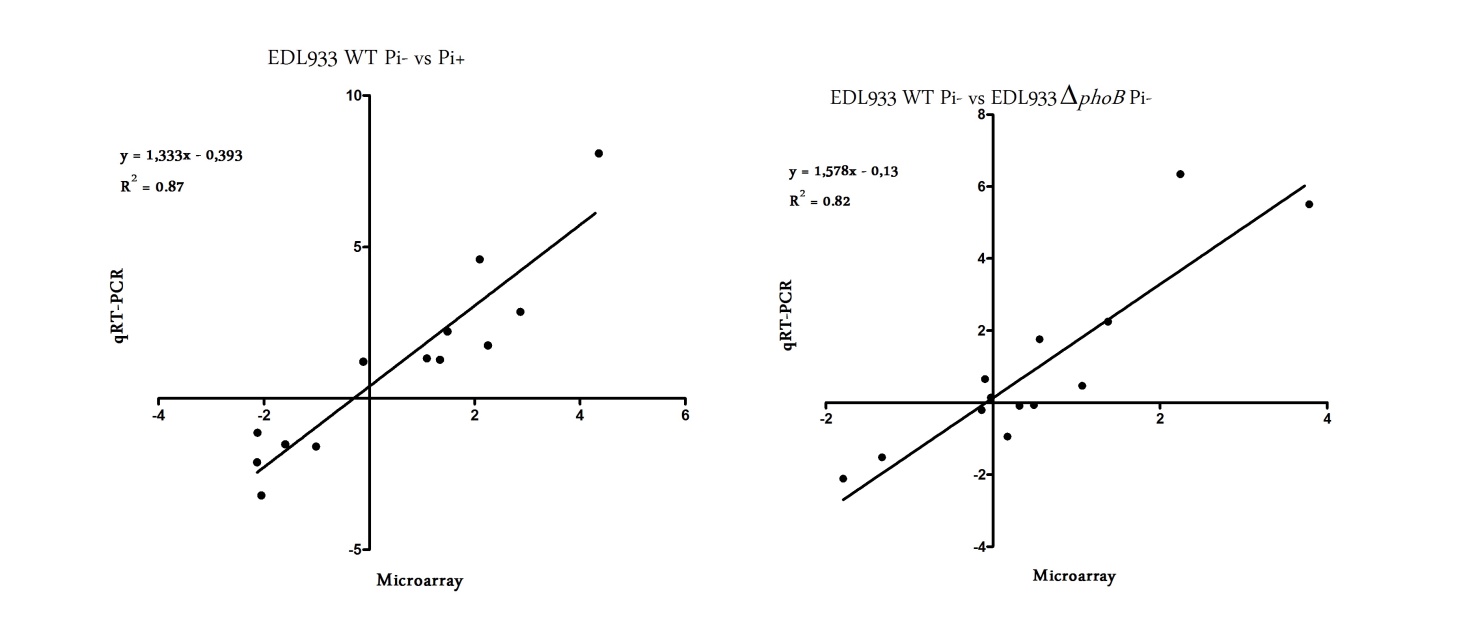

**Figure S2**. Microarray results were validated by triplicate qRT-PCR on 13 representative genes. Each reaction was normalized to *tus* gene and the variation rate was calculated using 2^-ΔΔCt^ method.
