# Supplementary material for: PhoB Activates Escherichia coli O157:H7 Virulence Factors in Response to Inorganic Phosphate Limitation
Source: PLoS One. 2014 Apr 7;9(4):e94285. doi: 10.1371/journal.pone.0094285 (PMC3978041; doi:10.1371/journal.pone.0094285)
Supplement: Table S1 — Primers used in this study. (DOCX) [file pone.0094285.s003.docx]

**Table S1.** Primers used in this study

| **Primers** | **Sequences 5’ – 3’** |
| --- | --- |
| ***stx2* promoter fusion** | |
| stx2-T7polCam-F  stx2-T7polCam-R | CTGCGCCGGGTCTGGTGCTGATTACTTCAGCCAAAAGGAACACCTGTATATGCACACGATTAACATCGC  GTGACACAGATTACACTTGTTACCCACATACCACGAATCAGGTTATGCCATGGAGTTCTGAGGTCATTACTG |
| **Probes for EMSA** | |
| P_LEE1_- For-6Fam  P_LEE1_-Rev  P_LEE2_- For-6Fam  P_LEE2_-Rev  P_LEE3_- For-6Fam  P_LEE3_-Rev  P_stx2_- For-6Fam  P_stx2_-Rev | GCTGAATGTATGGACTTGTTGT  TGTTAACGAGATGATTTTCTTCT  AATCTTAAAAACTCTTCAACGT  CTCATCCACTGAGTTATTTCCA  TTGCCTATGGGATAATTTGGTT  CTCATCCACTGAGTTATTTCCA  TAGTCAGTCAGAACGGATGAT  ACAGGTGTTCCTTTTGGCTGA |
