# Supplementary material for: PhoB Activates Escherichia coli O157:H7 Virulence Factors in Response to Inorganic Phosphate Limitation
Source: PLoS One. 2014 Apr 7;9(4):e94285. doi: 10.1371/journal.pone.0094285 (PMC3978041; doi:10.1371/journal.pone.0094285)
Supplement: Table S2 — Functional classification of upregulated genes of microarray data comparing the wild-type strain grown low Pi to in high Pi (Pi-dependent) and comparing the wild-type strain to Δ phoB mutant both grown low Pi (PhoB-dependent). (DOCX) [file pone.0094285.s004.docx]

**Tables S2.** Functional classification of upregulated genes of microarray data comparing the wild-type strain grown low Pi to in high Pi (Pi-dependent) and comparing the wild-type strain to Δ*phoB* mutant both grown low Pi (PhoB-dependent).

| **Gene or Operon** | **Function and description** | | **Microarray Fold Change ^a^** | | | |
| --- | --- | --- | --- | --- | --- | --- |
|  |  |  | **Pi-dependent** | | **PhoB-dependent** | |
| **Transcription & regulatory functions** | | |  | |  | |
| phoB-R  phoU  gadE  Z2382  adiY  ykgA  Z0332  Z2510  narL  csgDE  rpoH  dhaR  yegE  yjcT  ygeV  uhpA  hns  ECs1091 | DNA-binding response regulator in TCS with PhoR (or CreC)  negative regulator of PhoBR  DNA-bindingtranscriptional activator /acid-responsive regulator of gadA and gadBC  Putative antitermination protein  Putative ARAC-type regulatory protein  Putative AraC-like transcriptional regulator  Ogr family transcription activator encoded in prophage CP-933I (OI #08)  Putative transcriptional repressor (OI #70)  transcriptional regulator  DNA-binding transcriptional activator for *csgBA*  RNA polymerase factor sigma-32  DNA-binding transcription activator of the *dhaKLM* operon  Predicted diguanylate cyclase, GGDEF domain signaling protein  D-allose kinase; NAGC-like transcriptional regulator  Sigma-54-dependent transcriptional regulator  DNA-binding response regulator in TCS wtih UhpB  Global DNA-binding transcriptional dual regulator H-NS  putative transcriptional regulator (pchA) | | 4,28  12,27  7,32  2,25  2,81  2,15  2,34  2,25  2,15  2,20  --  --  --  --  --  --  --  7,55 | | 13,81  8,56  2,09  --  --  --  --  --  --  --  2,61  2,19  2,01  2,07  2,26  2,46  2,56  -- | |
| **Transport & binding** | | |  | |  | |
| pstSCAB  phoE  ugpBAECQ  napA  ftnA  xylF  Z5691  Z5689  galP  tnaB  artJ  focA  fecE | High-affinity phosphate ABC transporter  Outer membrane phosphoporin protein E  Glycerol-3-phosphate transporter  Nitrate reductase catalytic subunit  Ferritin iron storage protein (cytoplasmic)  D-xylose transporter subunit  Putative ATP-binding component of sugar ABC transporter  Putative periplasmic ribose-binding protein of ABC transport system  D-galactose transporter  Tryptophan permease TnaB  Arginine 3rd transport system  Formate transporter  KpLE2 phage-like element; iron-dicitrate transporter | | 47,48  40,39  7,92  3,06  7,40  2,14  2,46  2,18  --  --  --  --  -- | | 15,67  11,72  5,67  --  --  --  -2,60  --  2,31  2,08  3,66  2,81  2,17 | |
| **Energy metabolism** | | |  | |  | |
| ykgJ  frdD  yliI  pflB  gpmA  gadA  gadBC  talB  pfkA  yqeA  ppsA  pgk  dld  glgA  cydA  tdh  ybhM | Putative ferredoxin  Fumarate reductase (anaerobic), membrane anchor subunit  Soluble aldose sugar dehydrogenase  Pyruvate formate lyase I  Phosphoglyceromutase 1  Glutamate decarboxylase alpha  Glutamate decarboxylase beta, glutamic acid:γ-aminobutyrate antiporter  Transaldolase B  6-phosphofructokinase  Carbamate kinase / predicted amino acid kinase  Phosphoenolpyruvate synthase  Phosphoglycerate kinase  D-lactate dehydrogenase  Glycogen synthase  Cytochrome D ubiquinol oxidase  Threonine 3-dehydrogenase, NAD(P)-binding  Conserved inner membrane protein | 2,06  2,09  2,09  3,29  2,18  6,96  10,46  2,18  2,35  2,17  2,69  --  --  --  --  --  2.09 | | --  --  --  --  --  --  2.41  --  --  -2,24  --  2,21  2,60  3,26  2,12  2,04  -2,22 | |  |
|  |  |  | |  | |  |
| **Central intermediary metabolism** | |  | |  | | |
| phnCDEFGHIJKLMNO  phoA- psiF  amn  gldA  gst  Z4209  adhE | Phosphonate transporter and metabolisme  Alkaline phosphatase/P-starvation-inducible prot  AMP nucleosidase  Glycerol dehydrogenase  Glutathionine S-transferase  Aspartate/ornithine carbamoyltransferase family protein  Bifunctional acetaldehyde-CoA/alcohol dehydrogenase | 81,45  131,70  14,54  2,41  2,50  2,34  -- | | | 11,61  58,70  5,24  --  --  -2,15  2.98 |  |
| **DNA metabolism** | | | | | | |
| mutH  ybfLD  ihfB  Z5187 | Methyl-directed mismatch repair protein  H repeat-containing protein / predicted transposase  Integration host factor subunit beta  Putative replicase | 2,07  2,60  2,62  3,01 | | | --  -2,30  --  -2,13 | |
| **Cell envelope** | |  | | |  | |
| pgaABCD  slp  Z0024  lpxC  yeaF  wzx  slyB  fcI  Z3198  wcaJ | Required for PGA synthesis; an adhesin essential in biofilm  OMP induced after carbon starvation  Putative type-1 fimbrial protein  UDP-3-O-acyl N-acetylglucosamine deacetylase  Scaffolding protein for murein synthesizing machinery  O antigen flippase  Putative OMP  fucose synthetase  GDP-D-mannose dehydratase  Predicted UDP-glucose lipid carrier transferase | | 4,57  2,85  2,32  --  --  --  --  -2,22  --  2,04 | | 2,40  2,75  -2,10  2.27  3,31  2,16  2,06  2,35  2,26  -2,45 | |
| **Cellular processes** | | |  | |  | |
| yggR  vgrE  ahpCF  uspF  uspA  uspD  slyA  Z4326  ddg | Predicted pilus retraction ATPase  Rhs element protein (OI #65)  Alkyl hydroperoxide reductase subunit C  Nucleotide binding protein in the class II universal stress protein family  Universal stress protein A , global response regulator  Universal stress protein UspD  Increases expression of HlyE by antagonizing the negative effects of H-NS  Putative enterotoxin  lipid A biosynthesis palmitoleoyl acyltransferase | | 2,50  4,47  6,28  3,48  4,37  3,55  2,59  --  -- | | -2,22  --  11,17  --  --  --  --  2,69  2,03 | |
| **Protein fate** | | | | |  | |
| yecA  sohB  dnaK  gspD  pepP | Conserved protein, UPF0149 family  Predicted inner membrane peptidase; multicopy suppressor of htrA(degP  Chaperone Hsp70, co-chaperone with DnaJ  General secretory pathway component, cryptic  Proline aminopeptidase P II | | 3,45  --  --  --  -- | | --  2,03  2,11  2,13  2,18 | |
| **Protein synthesis** | | |  | |  | |
| ycgV  yibD  Efa-1/Z4332  Z2257  arnA | Partial putative adhesion protein  Predicted glycosyl transferase  Putative cytotoxin, involved in posttranscriptional regulation of type III secreted prot  Rhs element protein  Predicted barnase inhibitor | | 4,39  80,87  3,10  2,07  -- | | --  14,99  2,29  --  2.02 | |
| **Amino acid biosynthesis** | | |  | |  | |
| ilvY  hisD  argCB | DNA-binding transcriptional dual regulator, positive regulator for ilvC  Histidinol dehydrogenase  Acetylglutamate  reductase- kinase | | 2,21  --  -- | | -2,43  2.26  2,53 | |
| **Fatty acid and phospholipids metabolism** | | |  | |  | |
| Z1139  fabF | Putative diacylglycerol kinase  3-oxoacyl-(acyl carrier protein) synthase II | | 2,21  -- | | --  2,37 | |
| **Purines, pyrimidines, nucleosides, and nucleotides** | | |  | |  | |
| ugd  Z3513 | UDP-glucose 6-dehydrogenase  Bifunctional UDP-glucuronic acid decarboxylase | | --  -- | | 2,52  2,02 | |
| **Biosynthesis of cofactors, prosthetic groups, and carriers** | | |  | |  | |
| moaE  dkgB  gshB  yggC | Molybdopterin guanine dinucleotide biosynthesis protein  2,5-diketo-D-gluconate reductase B  Glutathione synthetase  Conserved protein with nucleoside triphosphate hydrolase domain | | 2,30  8,92  --  -- | | --  --  2,05  2,24 | |
| **Viral, prophage functions, Mobile element functions (O islands, pathogenicity islands)** | | |  | |  | |
| xisW / Z1425  Z1426  Z1428  Z1432  gamW-exoW  Z1441  Z1442  Z1448  Z1449  Z1452  Z1453  ninG /Z1458  Z1459  Z1460  Ant / Z1471  Z1473  Z1483  Z1485  Z1493  Z1498  Z1501  xisN / Z1765  yafF / b1460  Z1782  Z1874  Z1916  Z2079  Z2122  Z6045  Z6065  ECs1648  Z0984  Z0954  Z3073  ydcC / b1460  Z1135  Z3354  Z3360  Z0953  Z0954  Z4313  ECs1229  Noh / b0560  Z3929  Z3938  Z3128  Z1326  intQ  Z1875  c_1462 | Putative excisionase for prophage BP-933W (OI #45)  unknown protein encoded by bacteriophage BP-933W (OI #45)  unknown protein encoded by bacteriophage BP-933W (OI #45)  unknown protein encoded by bacteriophage BP-933W (OI #45)  host-nuclease inhibitor protein Gam of bacteriphage (OI #45)  unknown protein encoded by bacteriophage BP-933W (OI #45)  putative antitermination protein N of bacteriophage BP-933W (OI #45)  regulatory protein Cro of bacteriophage BP-933W (OI #45)  putative regulatory protein CII of bacteriophage BP-933W (OI #45)  Unknown protein encoded by bacteriophage BP-933W (OI #45)  Unknown protein encoded by bacteriophage BP-933W (OI #45)  Unknown protein encoded by bacteriophage BP-933W (OI #45)  Antitermination protein Q of bacteriophage BP-933W (OI #45)  unknown protein encoded by bacteriophage BP-933W (OI #45)  putative antirepressor protein Ant of bacteriophage BP-933W (OI #45)  putative endopeptidase Rz of bacteriophage BP-933W (OI #45)  putative tail fiber protein of bacteriophage BP-933W (OI #45)  unknown protein encoded by bacteriophage BP-933W (OI #45)  unknown protein encoded by bacteriophage BP-933W (OI #45)  unknown protein encoded by bacteriophage BP-933W (OI #45)  unknown protein encoded by bacteriophage BP-933W (OI #45)  putative excisionase for prophage CP-933N (OI #50)  H repeat-containing protein  unknown protein encoded by prophage CP-933N (OI #50)  putative antiterminator Q of prophage CP-933X (OI #52)  putative tail component of prophage CP-933X and cryptic prophage CP-933P  unknown protein encoded in ISEc within CP-933O (OI #57)  putative holin protein of prophage CP-933O (OI #57)  putative terminase encoded by prophages CP-933N (OI #50) & CP-933P  unknown protein encoded by cryptic prophage CP-933P  putative tail fiber component J of prophages CP-933U (OI#79) & CP-933M (OI#44)  unknown protein encoded by prophage CP-933K  serine/threonin protein phosphatise encoded by prophage CP-933K (OI #36)  unknown protein encoded within prophage CP-933U (OI #79)  conserved protein  complement resistance protein  putative exclusion protein ren of prophage CP-933V (OI #93)  unknown protein encoded within prophage CP-933V  NinG / unknown protein encoded by prophage CP-933K (OI #36)  serine/threonin protein phosphatise encoded by prophage CP-933K (OI #36)  putative pathogenicity island integrase  putative tail fiber protein of bacteriophage BP-933W (OI #45)  putative DNA packaging protein of prophage CP-933X (OI #52)  unknown protein encoded by prophage CP-933Y (OI #108)  hypothetical protein (OI #108)  putative inhibitor of cell division encoded within prophage CP-933U (OI #79)  putative inhibitor of cell division encoded by cryptic prophage CP-933M (OI #44)  integrase fragment, cryptic prophage CP-933P/ intP_2  putative holin protein of prophage CP-933X  putative tail component of prophage CP-933V | | 4,80  3,63  3,05  3,16  3,35  4.98  4,79  4,76  2,62  3,22  2,05  2,79  2,21  3,08  3,33  11,63  2,48  3,26  3,35  4,00  3,16  2,11  2,98  4,96  2,02  2,11  2,39  2,44  2,23  2,21  3,16  2,04  2,28  2,49  2,98  2,89  2,92  2,02  2,03  2,28  2,27  3,66  3,62  3,09  2,67  2,68  2,09  2,23  2,06  -- | | --  --  --  --  --  --  --  --  --  --  --  --  --  --  --  --  --  --  --  --  --  --  --  --  --  --  -3,55  --  --  --  --  --  --  --  --  -2,27  --  --  --  --  --  --  --  --  -2,30  -3,41  -2,33  -2,51  --  2,52 | |
| Z3269  hopD/ Z4693  Z4883  Ler / Z5140  orf2 (escE)  cesAB/ Z5138  orf4// Z5137  orf5/ Z5136  sepD/Z5125  escL/ Z5121  escV/ Z5120  escN / Z5119  orf15/Z5118  orf16/Z5117  espH/Z5115  cesF/Z5114  map/ Z5113  tir /Z5112  eae/ Z5110  escD/Z5109  sepL/Z5108  espD/Z5106  espB//Z5105  escF/Z5103  espF/Z5100  nleB/ Z4328  nleE/ Z4329  nleH Z6021  nleG-1/ Z2149 | Protein of Unknown function encoded in (OI #89)  putative leader peptidase (OI #130)  HicA-like protein (OI #139)  LEE regulator encoded in lee1 operon (OI #148)  LEE-encoded protein in lee1 operon (OI #148)  LEE-encoded protein in lee1 operon (OI #148)  LEE-encoded protein in lee1 operon (OI #148)  LEE-encoded protein in lee1 operon (OI #148)  LEE-encoded protein in lee2 operon (OI #148)  LEE-encoded protein in lee3 operon (OI #148)  LEE-encoded T3SS membrane-associated ATPase, lee3 operon (OI #148)  LEE-encoded T3SS ATPase, lee3 operon (OI #148)  LEE-encoded protein in lee3 operon (OI #148  LEE-encoded protein in lee3 operon (OI #148)  LEE-encoded effector translocated to the host cell membrane by the T3SS, lee3 operon (OI#148)  LEE-encoded chaperone (OI #148)  Type III secreted protein (OI #148)  translocated intimin receptor protein lee5 operon (OI #148)  Gamma intimin adherence protein lee5 operon (OI #148)  [LEE-encoded T3SS component](http://www.ncbi.nlm.nih.gov/gene/8219239)  (OI #148)  LEE-encoded T3SS component, lee4 operon (OI #148)  LEE-encoded secreted protein EspD, lee4 operon (OI #148)  LEE-encoded secreted protein EspB, lee4 operon (OI #148)  EscF / hypothetical protein, lee4 operon (OI #148)  EspF / hypothetical protein, lee4 operon (OI #148)  Non-LEE-encoded effector protein (OI #122)  Non-LEE-encoded effector protein (OI #122)  Non-LEE-encoded effector protein (OI #71)  Non-LEE-encoded effector protein encoded in CP-933O, colonization factor (OI #57) | | 3,31  2,04  2,01  2,80  3,25  3,03  3,50  4,58  --  --  --  --  --  2,42  2,42  --  --  --  --  2,17  --  --  2,13  --  2,02  --  --  --  -- | | --  -2,17  --  --  --  --  --  --  2,25  3,32  2,15  2,91  3,35  2,56  3,41  2,07  2,18  2.50  2,36  2,37  4,11  2,18  --  2,45  2,28  2,97  2,53  2,56  2,02 | |
| **Non-coding RNA** | | | | |  | |
| Ffs / b0455  csrC / b4457  ssrS / b2911  rybA/ b4416  gcvB / b4443  ryjA/ b4459  rnpB/ b3123  gad/ b4452  rybB/ b4417 | 4.5S RNA component of the signal recognition particle (SRP)  RNA inhibitor of CsrA  6S RNA  sRNA required for Mn homeostasis, under peroxide stress  Regulatory sRNA requires Hfq and regulates expression of genes e.g. csgD  RyjA small RNA  catalytic subunit of RNAse P  GadY small regulatory RNA  RybB small regulatory RNA | | 11,99  4,21  3,59  2,90  2,76  2,43  --  --  -- | | 2,19  --  --  --  --  2,40  4,90  2,90  2,32 | |

^a^ :(--) represents the not-significant changes.
