# Supplementary material for: PhoB Activates Escherichia coli O157:H7 Virulence Factors in Response to Inorganic Phosphate Limitation
Source: PLoS One. 2014 Apr 7;9(4):e94285. doi: 10.1371/journal.pone.0094285 (PMC3978041; doi:10.1371/journal.pone.0094285)
Supplement: Table S3 — Functional classification of downregulated genes of microarray data comparing the wild-type strain grown low Pi to in high Pi (Pi-dependent) and comparing the wild-type strain to Δ phoB mutant both grown low Pi (PhoB-dependent). (DOCX) [file pone.0094285.s005.docx]

**Tables S3.** Functional classification of downregulated genes of microarray data comparing the wild-type strain grown low Pi to in high Pi (Pi-dependent) and comparing the wild-type strain to Δ*phoB* mutant both grown low Pi (PhoB-dependent).

| **Gene/**  **operon** | **Pi-dependent** | | | | **PhoB-dependent** | **Function and description ^a^** | **Gene/**  **operon** | **Pi-dependent** | | | **PhoB-dependent** | | **Function and description ^a^** |  |
| --- | --- | --- | --- | --- | --- | --- | --- | --- | --- | --- | --- | --- | --- | --- |
| **Transcription & regulatory functions** | | | | | | | | | | | | | |  |
| rpoA | -6,33 | | | | -- | DNA-directed RNA polymerase subunits | rhlE | -3,80 | | | -- | | ATP-dependent RNA helicase |  |
| rpoBC | -7,06 | | | | -- |  | dbpA | -3,48 | | | -- | |  |  |
| rpoD | -2,63 | | | | -- |  | deaD | -2,88 | | | -- | |  |  |
| rpoZ | -3,46 | | | | -- |  | pnp | -4,00 | | | -- | | Polynucleotide phosphorylase/polyadenylase |  |
| rnc | -3,52 | | | | -- | Ribonucleases | cusR | -2,41 | | | -- | | Two-Component Signal Transduction System; DNA-binding response regulator and/or sensor kinase |  |
| rnpA | -14,81 | | | | -- |  | phoPQ | -2,11 | | | -- | |  |  |
| rnb | -3,14 | | | | -- |  | basRS | -2,35 | | | -- | |  |  |
| rnhB | -5,41 | | | | -- |  | yehU | -4,48 | | | -- | |  |  |
| emrR | -3,23 | | | | -- | DNA-binding transcriptional repressor | glnLG | -9,01 | | | -- | |  |  |
| purR | -5,55 | | | | -- |  | cstA | -2,14 | | | -- | | Carbon starvation protein |  |
| betI | -3,46 | | | | -- |  | yjiY | -2,31 | | | -- | |  |  |
| dgsA | -3,00 | | | | -- |  | cpdA | -2,89 | | | -- | | 3',5' cAMP phosphodiesterase |  |
| greA | -3,40 | | | | -- | Transcription antitermination factors | cyaA | -2,19 | | | -- | | Adenylate cyclase |  |
| nusA | -2,70 | | | | -- |  | malT | -2,58 | | | -- | | Transcriptional regulator |  |
| nusG | -2,07 | | | | -- |  | pdhR | -2,16 | | | -- | |  |  |
| uhpC | -2,01 | | | | -- | Membrane protein regulates uhpT expression | lrp | -2,16 | | | -- | |  |  |
| rbfA | -2,74 | | | | -- | Ribosome-binding factor | ispH | -2,16 | | | -- | | Diphosphate reductase |  |
| rnc | -3,51 | | | | -- | RNase III/ role in degradation of RNA | mhpR | -- | | | -2,10 | | Transcriptional activator, 3HPP-binding |  |
| fimZ | -- | | | | -3,32 | Transcriptional regulator involved in fimbrial expression (LuxR/UhpA family) | yidL | -- | | | -2,15 | | Predicted transcriptional regulator, AraC family |  |
| srmB | -- | | | | -2,85 | ATP-dependent RNA helicase SrmB | ycjW | -- | | | -2,03 | | Putative LACI-type transcriptional regulator |  |
| ybbS | -- | | | | -2,06 | Transcriptional activator of the allD operon | ydeO | -- | | | -2,25 | | Transcriptional activator for mdtEF |  |
| molR_ABD | -- | | | | -3,89 | Putative regulators (fragment) |  |  | | |  | |  |  |
| **Transport & binding** | | | | | | | | | | | | | |  |
| livHMG | -4,02 | | | | -- | Branched-chain amino acid transporter | aroP | -6,44 | | | -- | | Aromatic amino acid transporter |  |
| cysPUWA | -3,23 | | | | -- | Sulfate / thiosulfate ABC transporter | exbBD | -3,16 | | | -- | | Biopolymer transport |  |
| ptsG | -3,57 | | | | -- | PTS system glucose-specific transporter (glucose PTS permease) | glpFK | -4,51 | | | -- | | Glycerol uptake channel and kinase |  |
| ptsH | -2,16 | | | | -- | Hpr a non-specific protein of the phosphoenolpyruvate dependent PTS | gltJKL | -2,60 | | | -- | | Glutamate and aspartate transporter |  |
| hisJQMP | -2,89 | | | | -- | Histidine ABC transport | mglB | -3,80 | | | -- | | Galactose-binding transport protein |  |
| potABC | -4,90 | | | | -- | Putrescine / spermidine ABC transporter | manXY | -3,03 | | | -- | | PTS system, mannose-specific (mannose PTS permeases) |  |
| metNIQ | -2,61 | | | | -- | DL-methionine transporter | lysP | -5,45 | | | -- | | Lysine transporter |  |
| msbA-lpxK | -2,73 | | | | -- | Lipopolysaccharide ABC transporter | fepC | -3,37 | | | -- | | Iron-enterobactin transporter ATP-binding protein |  |
| artPQM | -3,99 | | | | -- | Arginine ABC transporter | putP | -2,75 | | | -- | | Sodium/proline symporter |  |
| btuB | -3,58 | | | | -- | Vitamin B12/cobalamin outer membrane transporter | gsiA | -2,09 | | | -- | | Glutathione transporter |  |
| dctA | -3,45 | | | | -- | C4-dicarboxylate transporter | dppB | -3,01 | | | -- | | Dipeptide transporter |  |
| rbsDB | -4,14 | | | | -- | D-ribose transporter | mtr | -2,78 | | | -- | | Tryptophan transporter of high affinity |  |
| cusCFBA | -5,89 | | | | -- | Copper / iron or silver efflux transport system | malEF | -2,46 | | | -2,14 | | Maltose ABC transporter |  |
| copA | -3,48 | | | | -- |  | lamB | -3,89 | | | -- | |  |  |
| trkA | -3,01 | | | | -- | K+ transporter | oppB | -2,26 | | | -- | | Oligopeptide transporter |  |
| trkH | -2,47 | | | | -- |  | lptCAB | -2,25 | | | -- | | Lipopolysaccharide transporter |  |
| yobA-yebZ | -2,48 | | | | -- | Putative resistance proteins | lolCD | -2,42 | | | -- | | Outer membrane-specific lipoprotein transporter |  |
| yjjK | -3,41 | | | | -- | Putative ABC transporter ATP-binding protein | ydjN | -2,80 | | | -- | | Predicted transporter /// part of a kinase |  |
| yheS | -2,29 | | | | -- |  | fadL | -2,66 | | | -2,38 | | Long-chain fatty acid outer membrane transporter |  |
| yddAB | -3,82 | | | | -- |  | Z0463 | -2,05 | | | -- | | Hexosephosphate transport; putative response regulator |  |
| ptsA | -- | | | | -3,03 | PEP-protein phosphotransferase system enzyme I | fepB | -- | | | -2,62 | | iron-enterobactin transporter |  |
| fucP | -- | | | | -2,11 | L-fucose transporter | agaV | -- | | | -2,33 | | PTS system N-acetylgalactosamine-specific transporter |  |
| ygfU | -- | | | | -2,26 | Putative purine permease | frvA | -- | | | -2,23 | | Putative fructose-like phosphotransferase system |  |
| afuA | -- | | | | -2,02 | Periplasmic ferric iron-binding protein | nanT | -- | | | -2,40 | | sialic acid transporter |  |
| yabJ | -- | | | | -2,01 | Thiamin transporter subunit | yjfF | -- | | | -3,21 | | Inner membrane ABC transporter permease |  |
| nikA | -- | | | | -2,13 | Periplasmic binding protein for nickel | Z5690 | -- | | | -2,20 | | Putative permease of ribose ABC transport system |  |
| Z2240 | -- | | | | -2,47 | IpaH-like protein | Z3023 | -- | | | -2,09 | | Putative secreted protein |  |
| **Energy metabolism** | | | | | | | | | | | | | |  |
| cyoABCDE | -7,48 | | | | -- | Cytochrome o ubiquinol oxidase – heme O synthase | gcvTHP | -5,68 | | | -- | | Glycine cleavage system |  |
| sucA | -2,20 | | | | -- | 2-oxoglutarate dehydrogenase, thiamin-requiring | speA | -2,82 | | | -- | | Arginine decarboxylase |  |
| nuoABCEFGHIJKLM | -4,05 | | | | -- | NADH dehydrogenase | atpIBEFHAGD | -4,30 | | | -- | | ATP synthase F0F1 complex |  |
| gpsA | -4,07 | | | | -- |  | fdx | -2,40 | | | -- | | Reduced ferredoxin, electron carrer protein |  |
| pntAB | -3,25 | | | | -- | NAD(P) transhydrogenase / pyridine nucleotide transhydrogenase | glpG | -2,24 | | | -- | | Intramembrane serine protease |  |
| sdhCDAB | -10,68 | | | | -- | Succinate dehydrogenase | lpdA | -2,58 | | | -- | | Lipoamide dehydrogenase |  |
| aceEF | -4,60 | | | | -- | Pyruvate dehydrogenase | glgX | -2,52 | | | -- | | Glycogen debranching enzyme |  |
| ppc | -3,80 | | | | -2,05 | Phosphoenolpyruvate carboxylase | maeA | -2,31 | | | -- | | Malate dehydrogenase |  |
| acs | -3,23 | | | | -- | Acetyl-CoA synthetase | gltA | -2,80 | | | -- | | Type II citrate synthase |  |
| etp | -4,41 | | | | -- | Phosphotyrosine-protein phosphatase | fdoG | -2,15 | | | -- | | Formate dehydrogenase |  |
| wzxE | -2,58 | | | | -- | Lipid III flippase, putative cytochrome | fldA | -2,25 | | | -- | | Flavodoxin |  |
| poxB | -- | | | | -2,08 | Pyruvate dehydrogenase | kdgK | -- | | | -2,58 | | 2-dehydro-3-deoxygluconokinase |  |
| adiA | -- | | | | -2,10 | Biodegradative arginine decarboxylase | ydiST | -- | | | -2,65 | | Predicted oxidoreductase ydiS  Ferredoxin-like protein ydiT, yfhL and |  |
| yihT | -- | | | | -2,18 | Predicted aldolase | yfhL | -- | | | -2,16 | |  |  |
| yhjN | -- | | | | -2,22 | Regulator of cellulose synthase, cyclic di-GMP binding | yeiI | -- | | | -2,35 | | Predicted kinase |  |
| sdaB | -- | | | | -2,06 | L-serine dehydratase | ynfF | -- | | | -2,43 | | S- and N-oxide reductase |  |
| ycbP | -- | | | | -2,37 | NAD(P)H-dependent FMN reductase | araAD | -- | | | -2,07 | | L-arabinose isomerise and L-ribulose-5-phosphate 4-epimerase |  |
| talc | -- | | | | -2,59 | Fructose-6-phosphate aldolase | glpD | -- | | | -2,16 | | Glycerol-3-phosphate dehydrogenase |  |
| hyfA | -- | | | | -2,11 | Hydrogenase 4 Fe-S subunit | glpC | -- | | | -4,04 | |  |  |
| treC | -- | | | | -2,13 | Trehalose-6-P hydrolase | hyaA | -- | | | -2,06 | | Hydrogenase-1 small subunit |  |
| treA | -- | | | | -2,04 | Periplasmic trehalase | hypE | -- | | | -2,26 | | Hydrogenases maturation proteins |  |
| ygeX | -- | | | | -2,14 | Diaminopropionate ammonia-lyase | fucK | -- | | | -2,19 | | L-fuculokinase |  |
| lacA | -- | | | | -2,83 | Galactoside O-acetyltransferase | srlR | -- | | | -3,70 | | Repressor SrlR regulator for gut (srl), glucitol operon |  |
| putA | -- | | | | -2,11 | Fused transcriptional regulator/proline andpyrroline-5-carboxylate dehydrogenase | astD | -- | | | -2,01 | | Succinylglutamic semialdehyde dehydrogenase |  |
| malS | -- | | | | -2,78 | Periplasmic alpha-amylase precursor | nrfG | -- | | | -2,53 | | Formate-dependent nitrite reductase complex subunit NrfG |  |
| ascB | -- | | | | -2,16 | Cryptic 6-phospho-beta-glucosidase | yihR | -- | | | -2,65 | | Putative aldose-1-epimerase |  |
| ebgA | -- | | | | -2,04 | Cryptic beta-D-galactosidase subunit alpha | yjeS | -- | | | -2,40 | | Predicted Epoxyqueuosine reductase |  |
| tktB | -- | | | | -2,43 | Transketolase | cadA | -- | | | -2,06 | | Lysine decarboxylase, acid-inducible |  |
| Z5618 | -- | | | | -3,10 | Sorbitol-6-phosphate 2-dehydrogenase |  |  | | |  | |  |  |
| **Central intermediary metabolism** | | | | | | | | | | | | | |  |
| ureDABCE | -2,90 | | | | -- | Ureases accessory and structural proteins | cysJI | -8,35 | | | -- | | Sulfite reductase |  |
| rsxABCGE | -2,77 | | | | -- | SoxR-reducing complex | gltBD | -5,67 | | | -- | | Glutamate synthase |  |
| cysDNC | -5,44 | | | | -- | Sulfate adenylyltransferases (cysDN) - adenylylsulfate kinase (cysC) | suhB | -2,30 | | | -- | | Inositol monophosphatase |  |
| codA | -3,79 | | | | -- | Cytosine deaminase | ygfT | -- | | | -2,52 | | Putative oxidoreductase Fe-S binding subunit |  |
| **DNA metabolism** | | | | | | | | | | | | | |  |
| rep | -3,08 | | | | -- | ATP-dependent DNA helicase Rep | gyrB | -3,10 | | | -- | | DNA gyrase subunit B |  |
| holC- valS | -4,12 | | | | -- | DNA polymerase III subunit chi - valyl-tRNA synthetase | rsmB | -3,42 | | | -- | | 16S rRNA methyltransferase B |  |
| priB | -26,77 | | | | -- | Primosomal replication protein N | parC | -2,14 | | | -- | | DNA topoisomerase IV subunit A |  |
| dnaAN-recF | -2,72 | | | | -- | Chromosomal replication initiation protein - DNA polymerase III subunit beta - recombination protein F | xerD | -2,52 | | | -- | | Site-specific tyrosine recombinase |  |
|  |  |  |  |  |  |  | recO | -2,24 | | | -- | |  |  |
| nth | -3,06 | | | | -- | Endonuclease III | dam | -2,64 | | | -- | | DNA adenine methyltransferase |  |
| recQ | -2,01 | | | | -- | ATP-dependent DNA helicase | dnaG | -2,99 | | | -- | | DNA primase |  |
| recG | -3,02 | | | | -3,46 |  | uvrC | -2,45 | | | -- | | Excinuclease ABC subunit C |  |
| holD | -4,31 | | | | -- | DNA polymerase III subunit psi | dnaE | -2,24 | | | -- | | DNA polymerase III subunit alpha |  |
| dnaB | -6,28 | | | | -- | Replicative DNA helicase | radA | -2,91 | | | -- | | DNA repair protein |  |
| holA | -3,32 | | | | -- | DNA polymerase III subunit delta | topA | -2,69 | | | -- | | DNA topoisomerase I |  |
| mutY | -5,45 | | | | -2,19 | Adenine DNA glycosylase | xseB | -2,69 | | | -- | | Exonuclease VII small subunit |  |
| metK | -3,19 | | | | -- | Methionine adenosyltransferase | gidB | -2,39 | | | -- | | 16S rRNA m7G527 methyltransferase |  |
| fis | -10,46 | | | | -- | Fis DNA-binding transcriptional dual regulator | ruvC | -3,34 | | | -- | | Component of RuvABC resolvasome, endonuclease |  |
| ybhP | -- | | | | -2,02 | Conserved protein, endo/exonuclease/phosphatase family PFAM PF03372 | holB | -4,19 | | | -- | | DNA polymerase III subunit delta' |  |
| ybgA | -- | | | | -2,05 | Conserved protein, DUF1722 family | yhdJ | -- | | | -2,33 | | DNA adenine methyltransferase, SAM-dependent |  |
| polB | -- | | | | -2,92 | DNA polymerase II | dinD | -- | | | -2,30 | | DNA-damage-inducible protein D |  |
| **Cell envelope** | | | | | | | | | | | | | |  |
| murA | -3,54 | | | | -- | UDP-N-acetylglucosamine enolpyruvoyl transferase | mepA | -2,86 | | | -- | | Murein DD-endopeptidase |  |
| mrcA | -2,53 | | | | -- | Peptidoglycan biosynthesis | mltC | -4,04 | | | -- | | Murein transglycosylase |  |
| mreBCD | -2,47 | | | | -- |  | asd | -2,31 | | | -- | | Aspartate-semialdehyde dehydrogenase |  |
| murFXDGC | -3,76 | | | | -- |  | nlpA | -2,25 | | | -- | | Cytoplasmic membrane lipoprotein-28 |  |
| waaFCL | -2,38 | | | | -- | LPS biosynthesis enzyme | fcI | -2,22 | | | -- | | Fucose synthetase |  |
| waaQGP | -2,91 | | | | -- | LPS core biosynthesis protein | dacB | -2,44 | | | -- | | D-alanyl-D-alanine endopeptidase |  |
| wzzE | -3,14 | | | | -- | LPS biosynthesis protein (ECA) | fhuA | -4,37 | | | -2,51 | | Ferrichrome / phage / antibiotic outer membrane porin |  |
| rlpB | -4,07 | | | | -- | LPS-assembly lipoprotein | ddlB | -2,51 | | | -- | | D-alanine--D-alanine ligase |  |
| mrdAB | -2,42 | | | | -- | Cell shape; peptidoglycan synthetase; penicillin-binding protein 2 | Z2200 | -3,28 | | | -- | | Putative major fimbrial subunit |  |
| lnt | -4,26 | | | | -- | Apolipoprotein N-acyltransferase | Z2201 | -3,02 | | | -- | | Putative fimbrial chaperone protein |  |
| rffEDGHC | -3,40 | | | | -- | UDP-N-acetylglucosamine epimerase/mannosaminuronic acid dehydrogenase  dTDP- glucose dehydratase/pyrophosphorylase/fucosamine acetyltransferase | bamC | -3,56 | | | -- | | OMP Assembly Complex |  |
|  |  |  |  |  |  |  | bamA | -3,26 | | | -- | |  |  |
| lpxDAB | -2,73 | | | | -- | Acetylglucosamine acyltransferase | yqhH | -- | | | -2,27 | | Puter membrane lipoprotein, Lpp paralog |  |
| htrE | -- | | | | -2,52 | Putative outer membrane usher protein | flgE | -- | | | -2,31 | | Flagellar hook protein |  |
| hofC | -- | | | | -2,14 | Type IV pilin biogenesis protein | ybiP | -- | | | -2,04 | | Predicted hydrolase, inner membrane |  |
| ppdD | -- | | | | -2,89 | Putative major pilin subunit | Z0257 | -- | | | -2,22 | | Lipoprotein |  |
| Z5223 | -- | | | | -2,22 | Putative fimbrial chaperone |  |  | | |  | |  |  |
| **Cellular processes** | | | | | | | | | | | | | |  |
| cspG | -5,26 | | | | -- | Cold shock protein | tolQRA | | -2,79 | | -- | | Tol-Pal Cell Envelope Complex membrane spanning protein in TolA-TolQ-TolR complex |  |
| cspH | -8,41 | | | | -- |  |  |  |  |  |  |  |  |  |
| xerC | -3,03 | | | | -- | Site-specific tyrosine recombinase | mukFE | | -2,01 | | -- | | Chromosome condensins subunits |  |
| surE | -5,32 | | | | -- | Stationary phase survival protein | ybeX | | -4,66 | | -- | | Predicted ion transport protein |  |
| cvpA | -15,76 | | | | -- | Colicin V production protein | ubiB | | -2,62 | | -- | | 2-octaprenylphenol hydroxylase |  |
| ybhC | -3,02 | | | | -- | Acyl-CoA thioesterase, lipoprotein | rlmE | | -2,32 | | -- | | 23S rRNA methyltransferase |  |
| ftsW | -3,18 | | | | -- | Essential cell division proteins | cafA | | -2,42 | | -- | | Ribonuclease G |  |
| ftsY | -2,52 | | | | -- |  | aldA | | -2,62 | | -- | | Aldehyde dehydrogenase |  |
| ftsLI | -2,04 | | | | -- |  | soda | | -2,06 | | -- | | Superoxide dismutase |  |
| ftsN | -2,65 | | | | -- |  | ybeZ | | -2,02 | | -- | | Putative ATP-binding protein in pho regulon |  |
| flgN | -- | | | | -2,26 | Flagellar export chaperone for FlgK and FlgL | emrY | | -- | | -2,00 | | Multidrug resistance protein Y |  |
| fliK | -- | | | | -2,14 | flagellar hook-length control protein | fimG | | -- | | -2,06 | | Minor component of type 1 fimbriae |  |
| flgCJ | -- | | | | -2,24 | Flagellar basal body rod protein FlgC and rod assembly protein/muramidase | ampC | | -- | | -5,12 | | Beta-lactamase |  |
| fliLM | -- | | | | -2,22 | Flagellar basal body-associated protein FliL and motor switch protein FliM | Z2263 | | -- | | -2,69 | | Rhs element protein |  |
| **Protein fate** | | | | | | | | | | | | | |  |
| ycbZ | -3,84 | | | | -- | Putative ATP-dependent protease | pcm | | -2,36 | | -- | | Protein-L-isoaspartate O-methyltransferase |  |
| secY | -9,84 | | | | -- | Preprotein translocase subunit | bamB | | -2,40 | | -- | | Lipoprotein required for OM biogenesis |  |
| secB | -3,22 | | | | -- |  | surA | | -3,15 | | -- | | Peptidyl-prolyl cis-trans isomerase |  |
| secDF | -4,85 | | | | -- |  | prfC | | -3,40 | | -- | | Peptide chain release factor 3 |  |
| secG | -2,78 | | | | -- |  | yibP | | -2,16 | | -- | | EnvC murein hydrolase |  |
| hflKC | -3,19 | | | | -- | Modulators for HflB protease specific for phage lambda cII repressor | pqqL | | -3,37 | | -- | | Zinc protease |  |
| prc | -3,32 | | | | -- | Mutational suppressor of prc thermosensitivity, outer membrane lipoprotein | rsmF | | -4,62 | | -- | | 16S rRNA m5C1407 methyltransferase |  |
| pepA | -5,27 | | | | -- | Leucyl aminopeptidase | lon | | -3,00 | | -- | | DNA-binding, ATP-dependent protease La |  |
| pepQ | -2,02 | | | | -- | Proline dipeptidase | tig | | -3,58 | | -- | | Trigger factor; a molecular chaperone involved in cell division |  |
| ylbB | -2,39 | | | | -- | Allantoate amidohydrolase | groS | | -2,84 | | -- | | Co-chaperonin GroES |  |
| dsbC | -2,37 | | | | -- | Protein disulfide isomerase | ddpX | | -2,64 | | -- | | D-alanyl-D-alanine dipeptidase |  |
| hslV | -2,36 | | | | -- | ATP-dependent protease peptidase | ybiY | | -- | | -2,24 | | Putative pyruvate formate-lyase 3 activating enzym |  |
| yraI | -- | | | | -2,34 | Predicted periplasmic pilin chaperone | yfcS | | -- | | -2,02 | | Putative fimbrial chaperone |  |
| hybD | -- | | | | -2,34 | Hydrogenase 2 maturation endopeptidase | yhbU | | -- | | -2,97 | | Putative collagenase |  |
| **Protein synthesis** | | | | | | | | | | | | | |  |
| rplNXEFRO-rpsNHE-rpmDJ | | -7,96 | | | 2,01 | 50S ribosomal subunit protein; L14, L24, L5, L6, L18, L15, S14, S8, S5, L30, L36 | rpsLG-fusA-tufA | | | -16,74 | | 2,53 | 30S ribosomal subunit protein; S12, S7- Elongation factor G – Elongation factor Tu |  |
| rpsP-rimM- trmD- rplS | | -15,96 | | | 2,72 | 30S ribosomal S16 protein - S16 rRNA-processing and G37 methyltransferase | rpsMKD-rplQ | | | -6,86 | | 2.15 | 30S ribosomal subunit protein; S13. S11, S4 50S ribosomal L17 |  |
| rpsJSCQrplCDWBVP-rpmC | | -33,60 | | | -- | 30S ribosomal subunit protein; S10, S19, S3, S17  50S ribosomal subunit protein; L3, L4, L23, L2, L22, L16, L29 | rplKAJL | | | -20,49 | | -- | 50S ribosomal subunit protein; L11, L1, L10, L7 |  |
|  |  |  |  |  |  |  | rpsA | | | -8,56 | | -- | 30S ribosomal subunit protein; S1 |  |
| rplU | | -2,36 | | | -- | 50S ribosomal subunit protein; L21, L27 | rpsU | | | -7,42 | | -- | 30S ribosomal protein S21 |  |
| rpsT | | -3,85 | | | -- | 30S ribosomal subunit protein; S20 | rplM- rpsI | | | -9,12 | | -- | 50S ribosomal subunit protein; L13- 30S S9 |  |
| rpsB- tsf | | -9,47 | | | -- | 30S ribosomal subunit protein; S2 - elongation factor EF-Ts | rpsFR-rplI | | | -24,38 | | -- | 30S ribosomal protein S6, S18- 50S L9 |  |
| rplY | | -7,87 | | | -- | 50S ribosomal protein L25 | rpmE | | | -8,98 | | -- | 50S ribosomal subunit protein L31 |  |
| rpsO | | -4,74 | | | -- | 30S ribosomal protein S15 | rpmE2 | | | -2,02 | | -- | 50S ribosomal protein L31 type B |  |
| rpmF | | -3,17 | | | -- | 50S ribosomal protein L32 | rumB | | | -3,22 | | -- | 23S rRNA m5U747 methyltransferase |  |
| rpmH | | -10,56 | | | -- | 50S ribosomal protein L34 | rumA | | | -3,23 | | -- | 23S rRNA m5U1939 methyltransferase |  |
| rpmBG | | -3,40 | | | -- | 50S ribosomal protein L28, L33 | metG | | | -2,92 | | -- | Methionyl-tRNA synthetase |  |
| pcnB | | -5,32 | | | -- | Poly(A) polymerase I | rluD | | | -3,36 | | -- | 23S rRNA pseudouridine synthase |  |
| pheST | | -6,48 | | | -- | Phenylalanyl-tRNA synthetase α- and β-chain | lysS | | | -2,19 | | -- | Lysine tRNA synthetase |  |
| alaS | | -3,26 | | | -- | Alanyl-tRNA synthetase | tyrS | | | -2,81 | | -- | Yrosyl-tRNA synthetase |  |
| spoT | | -4,02 | | | -- | Bifunctional (p)ppGpp synthetase and hydrolase | prfA | | | -2,60 | | -- | Peptide chain release factor RF-1 |  |
| queA | | -4,17 | | | -- | tRNA ribosyltransferase-isomerase | prfB | | | -3,94 | | -- | Peptide chain release factor RF-2 |  |
| relA | | -3,02 | | | -- | GDP/GTP pyrophosphokinase | argS | | | -2,25 | | -- | Arginyl-tRNA synthetase |  |
| yfiC | | -2,39 | | | -- | tRNA m6A37 methyltransferase | infA | | | -2,75 | | -- | protein chain initiation factor IF-1 |  |
| proS | | -2,03 | | | -- | Prolyl-tRNA synthetase | infB | | | -2,50 | | -- | Protein chain initiation factor IF2 |  |
| proQ | | -4,19 | | | -- | RNA chaperone, involved in posttranscriptional control of ProP levels | rsmA | | | -2,86 | | -- | 16S rRNA dimethyladenosine transferase |  |
| truB | | -3,07 | | | -- | tRNA pseudouridine synthase | hisS | | | -2,74 | | -- | Histidyl-tRNA synthetase |  |
| leuS | | -3,08 | | | -- | Leucyl-tRNA synthetase | cysS | | | -2,51 | | -- | Cysteinyl-tRNA synthetase |  |
| aspS | | -3,45 | | | -- | Aspartyl-tRNA synthetase | glyQ | | | -3,01 | | -- | Glycine tRNA synthetase |  |
| Tgt | | -6,06 | | | -- | tRNA-guanine transglycosylase | efp | | | -2,62 | | -- | Elongation factor P |  |
| miaA | | -2,97 | | | -- | tRNA(i6A37) synthase | rimI | | | -2,05 | | -- | Acetylase for 30S ribosomal subunit protein S18 |  |
| glnE | | -2,05 | | | -- | Glutamine synthetase adenylyltransferase | asnC | | | -2,28 | | -- | Asparaginyl-tRNA synthetase |  |
| pth | | -2,49 | | | -- | Peptidyl-tRNA hydrolase | ycdL | | | -- | | -2,23 | Predicted enzyme, isochorismatase homolog |  |
| yibK | | -- | | | -2,07 | Putative tRNA/rRNA methyltransferase YibK | trmA | | | -- | | -2,17 | tRNA (uracil-5-)-methyltransferase |  |
| yjcQ | | -- | | | -2,10 | Multidrug efflux system protein MdtO | yehI | | | -- | | -2,63 | Putative regulator |  |
| yhgE | | -- | | | -2,14 | Putative transport protein | yghZ | | | -- | | -2,46 | Aldo-keto reductase |  |
| thdF | | -- | | | -2,39 | tRNA modification GTPase TrmE | Z4504 | | | -- | | -2,22 | Disrupted fimbrial protein |  |
| rutB | | -- | | | -2,23 | isochorismatase family protein ycdL | Z3026 | | | -- | | -2,19 | Putative secreted protein |  |
| Z3058 | | -- | | | -2,24 | Putative OMP |  | | |  | |  |  |  |
| **Amino acid biosynthesis** | | | | | | | | | | | | | |  |
| trpLEDCB | | -9,96 | | | 2,48 | Tryptophan synthase operon | metE | | | -74,07 | | -- | Cobalamin-independent homocysteine transmethylase |  |
| hisCBHA | | -2,65 | | | -- | Biosynthesis of histidine | metH | | | -7,44 | | -- | Cobalamin-dependent methionine synthase |  |
| leuABCD | | -7,44 | | | -- | Isopropylmalate biosyntheses | cysE | | | -2,14 | | -- | Serine acetyltransferase |  |
| thrABC | | -4,67 | | | -- | Aspartokinase/homoserine dehydrogenase-kinase- threonine synthase | cysM | | | -6,75 | | -- | Cysteine synthase |  |
| aroC-mepA-yfcL | | -3,23 | | | -- | Chorismate synthase-murein endopeptidase | serB | | | -3,54 | | -- | 3-phosphoserine phosphatase |  |
| aroG | | -4,27 | | | -- | 2-dehydro-3-deoxyphosphoheptonate aldolases | asnA | | | -3,71 | | -- | Asparagine synthetase |  |
| aroF | | -12,85 | | | -- |  | asnB | | | -3,19 | | -- |  |  |
| ilvGMEDA | | -10,59 | | | -- | Valine, leucine and isoleucine biosyntheses | aroKB | | | -4,34 | | -- | Shikimate kinase- dehydroquinate synthase |  |
| ivbL-ilvBN | | -2,64 | | | -- |  | aroL | | | -3,45 | | -- |  |  |
| ilvC | | -49,34 | | | -- |  | lysC | | | -8,20 | | -- | Aspartate kinase |  |
| hyuA | | -- | | | 3,27 | Phenylhydantoinase |  | | |  | |  |  |  |
| **Fatty acid and phospholipids metabolism** | | | | | | | | | | | | | |  |
| pssA | | | | -2,77 |  | Phosphatidylserine synthase | lgt | | | -2,13 | | -- | Prolipoprotein diacylglyceryl transferase | |
| plsC | | | | -3,13 |  | 1-acylglycerol-3-phosphate O-acyltransferase | accA | | | -2,86 | | -- | Acetyl-CoA carboxyltransferase | |
| accBC | | | | -4,91 | -- | Biotin carboxyl carrier protein / acetyl-CoA and biotin carboxylase | pldB | | | -2,40 | | -- | Lysophospholipase L2 | |
| yceD-plsX-fabHDG | | | | -5,33 | 2,11 | Fatty acids biosynthesis | fabA | | | -3,42 | |  | 3-hydroxydecanoyl-[acp] dehydrase | |
| Z4853-Z4854 | | | | -2,17 |  | Putative acyl carrier proteins | cdsA | | | -2,87 | | -- | CDP-diglyceride synthase | |
| Arp | | | | -- | 2,62 | Regulator of acetyl CoA synthetase |  | | |  | |  |  | |
| **Purines, pyrimidines, nucleosides, and nucleotides** | | | | | | | | | | | | | |  |
| hflD-purB | | -5,96 | | |  | Lysogenization regulator- Adenylosuccinate lyase | gpt | | | -2,11 | | -- | Xanthine-guanine phosphoribosyltransferase |  |
| purEK | | -7,42 | | |  | N5-carboxyaminoimidazole ribonucleotide mutase and synthetase | ndk | | | -4,44 | | -- | Nucleoside di-P kinase |  |
| purHD | | -4,99 | | | -- | AICARtransformylase/IMP cyclohydrolase-Phosphoribosylamine-glycine ligase | cmk | | | -6,28 | | -- | Cytidylate kinase |  |
| purMN | | -13,72 | | | -- | Phosphoribosyl aminoimidazole synthetase and glycinamide Formyltransferase | prsA | | | -2,39 | | -- | Ribose-phosphate pyrophosphokinase |  |
| apt | | -3,24 | | | -- | Adenine phosphoribosyltransferase | pyrF | | | -2,39 | | -- | OMP decarboxylase |  |
| purC | | -5,15 | | | -- | Phosphoribosylaminoimidazole-succinocarboxamide synthase | pyrG | | | -2,54 | | -- | CTP synthetase |  |
| guaB | | -4,16 | | | -- | IMP dehydrogenase | upp | | | -2,69 | | -- | Uracil phosphoribosyltransferase |  |
| glmU | | -5,01 | | | -- | N-acetylglucosamine-1-P uridyltransferase/glucosamine-1-P acetyltransferase | gsk | | | -2,31 | | -- | Inosine-guanosine kinase |  |
| pyrD | | -4,90 | | | -- | Dihydroorotate dehydrogenase | pyrC | | | -2,20 | | -- | Dihydroorotase |  |
| purF | | -9,94 | | | -- | Amidophosphoribosyl transferase | pyrH | | | -3,30 | | -- | Uridylate kinase |  |
| trxB | | -3,61 | | | -- | Thioredoxin reductase | tmk | | | -3,19 | | -- | Thymidylate kinase |  |
| nrdA | | -4,71 | | | -- | Ribonucleoside diphosphate reductase | adk | | | -2,29 | | -- | Adenylate kinase |  |
| purT | | -3,00 | | | -2,17 | Phosphoribosylglycinamide formyltransferase | purU | | | -2,85 | | -- | Formyltetrahydrofolate deformylase |  |
| purL | | 3,84 | | | -- | Phosphoribosylformylglycinamide synthetase | udk | | | -2,06 | | -- | Uridine/cytidine kinase |  |
| nrdG | | -- | | | -2,91 | Anaerobic ribonucleotide reductase-activating protein | carAB | | | -9,95 | | -- | Carbamoyl phosphate synthase |  |
| nrdF | | -- | | | -3,70 | Ribonucleotide-di-P reductase subunit bet | gsk | | | -- | | -2,15 | inosine-guanosine kinase |  |
| **Biosynthesis of cofactors, prosthetic groups, and carriers** | | | | | | | | | | | | | |  |
| pncB | | -4,03 | | | -- | Nicotinate phosphoribosyltransferase | folE | | | -2,03 | | -- | GTP cyclohydrolase I |  |
| bioFC | | -5,44 | | | -- | Biotin biosynthesis , aminopelargonate biosynthesis | folK | | | -2,41 | | -- | 6-hydroxymethyl-7,8-dihydropterin pyrophosphokinase |  |
| thiL | | -3,26 | | | -- | Thiamin monophosphate kinase | folB | | | -2,15 | | -- | Dihydroneopterin aldolase |  |
| entCE | | -2,02 | | | -- | Isochorismate synthase EntC, MenF and enterobactin synthase subunit E | gshA | | | -2,52 | | -- | Glutamate-cysteine ligase |  |
| menF | | -3,38 | | | -- |  | ubiE | | | -2,11 | | -- | Ubiquinone/menaquinone biosynthesis methyltransferase |  |
| hemC | | -2,14 | | | -- | Hydroxymethylbilane synthase | pdxH | | | -2,08 | | -- | Pyridoxamine 5'-phosphate oxidase |  |
| ribH | | -2,53 | | | -- | Riboflavin synthase | ispU | | | -2,87 | | -- | Undecaprenyl pyrophosphate synthase |  |
| dxs | | -2,02 | | | -- | 1-deoxy-D-xylulose-5-phosphate synthase | prmC | | | -2,41 | | -- | Protein-(glutamine-N5) methyltransferase |  |
| yaeM | | -2,07 | | | -- | 1-deoxy-D-xylulose 5-phosphate reductoisomerase | nadR | | | -2,67 | | -- | Nicotinamide-nucleotide adenylyltransferase |  |
| metF | | -2,22 | | | -- | 5,10-methylenetetrahydrofolate reductase | pabB | | | -2,27 | | -- | Para-aminobenzoate synthase |  |
| ribF | | -2,30 | | | -- | Bifunctional riboflavin kinase/FMN adenylyltransferase | grxC | | | -2,42 | | -- | Reduced glutaredoxin 3 |  |
| hemA | | -2,31 | | | -- | Glutamyl-tRNA reductase | hemH | | | -- | | -2,69 | Ferrochelatase |  |
| nadB | | -- | | | -2,53 | L-aspartate oxidase | hemF | | | -- | | -2,11 | Coproporphyrinogen III oxidase |  |
| **Viral, prophage functions, mobile element functions (PAI)** | | | | | | | | | | | | | |  |
| Z0955 | | | -3,71 | | 2,39 | Unknown protein encoded by prophage CP-933K (OI #36) | Z1930 | | | -2,55 | |  | Putative protease encoded within prophage CP-933X (OI #52) |  |
| Z1337 | | | -2,23 | |  | Unknown protein encoded by cryptic prophage CP-933M (OI #44) | Z1643 | | | -3,05 | |  | Hypothetical protein (OI #48) |  |
| Z5899-Z5901 | | | -2,46 | |  | Putative ATP-dependent helicases (OI #172) | hfq | | | -2,34 | |  | HF-I, host factor for RNA phage Q beta replication |  |
| stx1AB | | | -4,14 | |  | Stx 1 subunit A and B encoded within prophage CP-933V (OI #93) | Z3943 | | | -- | | -2,60 | Putative enzyme; Integration, recombination (Prophage Related) |  |
| Z2039 | | | -- | | -5,39 | DicB, putative inhibitor of cell division encoded by cryptic prophage CP-933P | Z1852 | | | -- | | -2,20 | Putative holin protein of prophage CP-933C |  |
| ybiY | | | -- | | -2,24 | Putative pyruvate formate-lyase 3 activating enzyme | nlp | | | -- | | -3,25 | DNA-binding transcriptional regulator Nlp |  |
| Z3362 | | | -- | | -2,45 | Putative superinfection exclusion protein B of prophage CP-933V | yhbQ | | | -- | | -2.15 | GIY-YIG nuclease superfamily protein |  |
| **Non-coding RNA** | | | | | | | | | | | | | |  |
| sraB | -2,87 | | | | -- | Novel Novel sRNA, function unknown identified in a large scale screen | t44 | | | -15,23 | | -- | 30S ribosomal protein S2 |  |
| ryeE | -- | | | | -3,40 | sRNA that promotes degradation of the ompX, yqaE, nadE, and luxS mRNAs | sraG | | | -- | | -3,83 | PsrO small RNA |  |
| csrB | -- | | | | -3,60 | sRNA inhibits CsrA-mediated acceleration of glg mRNA degradation | oxyS | | | -- | | -2,92 | Oxidative stress regulator |  |
| micF | -- | | | | -2,44 | Represses production of OmpF in response to environmental stress conditions | SibB | | | -- | | -2,09 | sRNA (ryeD) |  |

^A^ : (--) represents the not-significant changes.
