# Supplementary material for: PhoB Activates Escherichia coli O157:H7 Virulence Factors in Response to Inorganic Phosphate Limitation
Source: PLoS One. 2014 Apr 7;9(4):e94285. doi: 10.1371/journal.pone.0094285 (PMC3978041; doi:10.1371/journal.pone.0094285)
Supplement: Table S4 — Pho Box matrix based on 12 Pho-Boxes sequences from the known Pho genes in EDL933 identified by Yuan et al . 2006. (DOCX) [file pone.0094285.s006.docx]

**Table S4**: Pho Box matrix based on 12 Pho-Boxes sequences from the known Pho genes in EDL933 identified by Yuan *et al*. 2006.

| **Position** | **1** | **2** | **3** | **4** | **5** | **6** | **7** | **8** | **9** | **10** | **11** | **12** | **13** | **14** | **15** | **16** | | **17** | **18** |
| --- | --- | --- | --- | --- | --- | --- | --- | --- | --- | --- | --- | --- | --- | --- | --- | --- | --- | --- | --- |
| *phoB* | C | T | G | T | C | A | T | A | A | A | T | C | T | G | A | C | | G | C |
| *pstS1* | G | T | G | T | C | A | T | C | A | A | A | C | T | G | T | C | | A | C |
| *pstS2* | C | T | G | T | C | A | C | A | T | T | C | C | T | T | A | C | | A | T |
| *pstS3* | C | T | T | A | C | A | T | A | T | A | A | C | T | G | T | C | | A | C |
| *phoA1* | C | T | G | T | C | A | T | A | A | A | G | T | T | G | T | C | | A | C |
| *phoA2* | C | T | T | T | T | C | A | A | C | A | G | C | T | G | T | C | | A | T |
| *phoE1* | C | T | G | T | A | A | T | A | T | A | T | C | T | T | T | A | | A | C |
| *phoE2* | A | T | A | T | C | A | T | T | A | A | T | C | T | G | T | A | | A | T |
| *ugpB1* | T | T | G | T | C | A | T | C | T | T | T | C | T | G | A | C | | A | C |
| *ugpB2* | C | T | A | T | C | T | T | A | C | A | A | A | T | G | T | A | | A | C |
| *ugpB3* | A | A | G | T | T | A | T | T | T | T | T | C | T | G | T | A | | A | T |
| *phnC* | C | T | G | T | T | A | G | T | C | A | C | T | T | T | T | A | | A | T |
| **Base pairs Frequency** | | | |  |  |  |  |  |  |  |  |  |  |  |  |  |  | |  |
| **A** | **16.7** | **8.3** | **16.7** | **8.3** | **8.3** | **83.4** | **8.3** | **58.3** | **33.3** | **75** | **25.0** | **8.3** | **0** | **0** | **25** | **41.7** | | **91.7** | **0** |
| **T** | **8.3** | **91.7** | **16.7** | **91.7** | **25** | **8.3** | **75.1** | **25** | **41.7** | **25** | **41.6** | **16.7** | **100** | **25** | **75** | **0** | | **0** | **41.7** |
| **C** | **66.7** | **0.0** | **0** | **0** | **66.7** | **8.3** | **8.3** | **16.7** | **25** | **0** | **16.7** | **75** | **0** | **0** | **0** | **58.3** | | **0** | **58.3** |
| **G** | **8.3** | **0.0** | **66** | **0** | **0** | **0** | **8.3** | **0** | **0** | **0** | **16.7** | **0** | **0** | **75** | **0** | **0** | | **8.3** | **0** |
